# Fake news detection: a survey of evaluation datasets

**DOI:** 10.7717/peerj-cs.518

**Published:** 2021-06-18

**Authors:** Arianna D’Ulizia, Maria Chiara Caschera, Fernando Ferri, Patrizia Grifoni

**Affiliations:** Institute of Research on Population and Social Policies, National Research Council, Rome, Italy

**Keywords:** Fake news detection, Online fake news, Evaluation datasets

## Abstract

Fake news detection has gained increasing importance among the research community due to the widespread diffusion of fake news through media platforms. Many dataset have been released in the last few years, aiming to assess the performance of fake news detection methods. In this survey, we systematically review twenty-seven popular datasets for fake news detection by providing insights into the characteristics of each dataset and comparative analysis among them. A fake news detection datasets characterization composed of eleven characteristics extracted from the surveyed datasets is provided, along with a set of requirements for comparing and building new datasets. Due to the ongoing interest in this research topic, the results of the analysis are valuable to many researchers to guide the selection or definition of suitable datasets for evaluating their fake news detection methods.

## Introduction

“Fake news is not simply false news. Its nature is determined by fraudulent content in news format as well as by an ability to travel as much as, and in some cases, even more than, authentic news”, as explained in UNESCO (2018) (https://unesdoc.unesco.org/ark:/48223/pf0000261065). This phenomenon is growing more and more with the digitalization of information and communication services. The European Commission report (2018) defines fake news as “all forms of false, inaccurate, or misleading information designed, presented and promoted to cause public harm intentionally or for profit”. The spread of fake news on the Internet represents a critical problem in today’s society. An increasing number of people rely on digital platforms for accessing news or exchanging information. Often, they do not have the necessary skill to distinguish between fake and real news. Helping people to detect fake news is becoming fundamental for avoiding negative influences on public opinion and their decisions. One of the most cited examples of how fake news can influence opinions is the United States presidential election in 2016. Several studies (Flynn, Nyhan & Reifler, 2017; Allcott & Gentzkow, 2017) demonstrated the significant influence that fake news exerted on the political debate, even to the extent of affecting the electoral outcomes.

In the past few years, the scientific community has shown a growing interest in fake news detection to mitigate the spread of false information and its negative influence on society. With this aim, several fake news detection techniques have been developed in attempts to automatically detect whether news is fake or not (Bondielli & Marcelloni, 2019). Most of these techniques formulate the issue of automatically detecting fake news as a supervised binary classification problem. Thus the news is categorized into two classes (fake or real news) and a dataset of labeled data is used to train and validate the classifier to achieve good performance (Dwivedi & Wankhade, 2021; Zhou & Zafarani, 2020). Therefore, the availability of a corpus of news articles labeled according to their level of veracity is a relevant problem for developing accurate fake news detection methods.

Collecting reliable datasets of fake and trustworthy news is not a trivial task. First of all, it requires the fact-checking of news to annotate items as true or false (or more rating levels according to the rating scale used). This process can be performed in four ways: by manual verification through expert-oriented fact-checking; by using computational tools (e.g., knowledge graphs and open web sources) for computational fact-checking; by crowd evaluation as in crowdsourced fact-checking; and by assessment sites for fact-checking. The former is a laborious and time-consuming process as it requires the manual annotation of news from the dataset by human annotators. The last three methods make fact-checking easier and faster as they rely on digital tools and the crowd for finding, reasoning, and analyzing the news content. However, computational fact-checking suffers from the inherent ambiguity of language that makes the detection less accurate, while crowdsourced fact-checking is less credible and accurate than expert-oriented fact-checking due to possible conflicting annotations. Therefore, a trade-off needs to be found to make the fact-checking process accurate, fast, and labor-saving. Moreover, having solved the issue of annotating the trustworthiness of news items, further issues emerge when collecting evaluation datasets for fake news detection. For instance, dataset developers have to decide on the size of the dataset, the variety of topics (e.g., societal, political, health, financial, etc.) and the media (e.g., texts, images, videos, etc.) of the collected news, and the type of disinformation (e.g., rumors, hoaxes, satire, etc.) addressed. According to the solutions provided for these issues, different datasets for fake news detection have been developed in the literature. These differ in terms of the news domain, application purpose, type of disinformation, language, size, news content, rating scale, spontaneity, and media platform. Analyzing and classifying them according to these features and comparing them according to different requirements (availability, verifiability, homogeneity, etc.) can provide practical benefits to researchers and practitioners dealing with fake news detection.

Although several surveys have been proposed in the last few years, dealing with various techniques applied to automatically detect fake news (detection techniques (Bondielli & Marcelloni, 2019), prediction techniques (Dutta et al., 2019), identification and mitigation techniques (Sharma et al., 2019), natural language processing techniques (Oshikawa, Qian & Wang, 2018), and detection and resolution techniques (Zubiaga et al., 2018)), to the best of our knowledge none of these surveys are focused on the evaluation of datasets for fake news detection. Some of them provide a section discussing existing datasets, but they do not detail their characteristics and the requirements to build new datasets. Therefore, the main contributions of the present survey are as follows: (1) to provide a Fake News Detection Datasets (FNDD) characterization composed of eleven characteristics extracted from the existing datasets; (2) to systematically review twenty-seven popular datasets for fake news detection based on the defined characterization; (3) to provide a set of requirements for comparing fake news detection datasets and for building new ones; (4) to provide a comparative analysis among them, and (5) to consolidate open challenges emerging from the quantitative and comparative analyses. Due to the ongoing interest in this research topic, the results of the analysis are valuable to many researchers to guide the selection or definition of suitable datasets for evaluating their fake news detection methods.

The paper is organized as follows. A brief overview of the existing definitions of “fake news” and current surveys dealing with fake news detection is provided in the “Related work” section. The “Survey methodology” section introduces the research methodology adopted to conduct the literature search and the analyses performed. The section on the “Characteristics of evaluation datasets used in fake news detection” reviews the main features of the datasets that have been used to analyze them quantitatively. The results of this analysis are presented in the “Quantitative analysis of surveyed datasets” section. In the “Comparative analysis of surveyed datasets” section, we compare the surveyed datasets considering several data requirements arising from the aforementioned characteristics. The “Open challenges” section presents the open challenges that resulted from the analyses performed. Finally, in the “Conclusion” section, we provide some concluding remarks.

## Related work

Despite the efforts reported in the literature, a general and shared definition of “fake news” has not yet been reached. One of the first definitions, widely adopted in recent studies, has been provided by Shu et al. (2017), who defined fake news as “a news article that is intentionally and verifiably false”. A similar definition has been proposed by Golbeck et al. (2018), that “fake news is information, presented as a news story that is factually incorrect and designed to deceive the consumer into believing it is true”. Both these definitions restrict the type of information to fake articles or stories, leaving out several different types of misleading information, like hoaxes, rumors, satire, click-bait, etc. Moreover, they restrict also the intent of deception to the dishonest intention to mislead consumers. Therefore, various authors have broadened the meaning of these definitions, proposing the following ones: “a news article or message published and propagated through media, carrying false information regardless [of] the means and motives behind it” (Sharma et al., 2019) and “all forms of false, inaccurate, or misleading information designed, presented and promoted to intentionally cause public harm or for profit” (European Commission, 2018). These two definitions capture a broader range of types of information and intents of deception. In this survey, we use the broader definition given by the European Commission, since it allows us to broadly include in our investigation all types of false information.

Given this notion of fake news, we provide a brief explanation to clarify what the task of fake news detection consists of. With this aim, we draw on the definition given by Sharma et al. (2019), which we simplify as follows: the task of fake news detection consists of predicting whether the news article is a piece of fake news or not. Since in this survey we use the broader definition of fake news, the task of fake news detection captures different types of application purposes, including rumor detection, clickbait detection, and veracity classification.

Fake news detection has been the topic of several surveys in the last few years. One of the first was published by Shu et al. (2017) with the aim of reviewing methods for detecting fake news on social media platforms, including a fake news characterization based on psychology and social theories, some evaluation metrics, and a list of four representative fake news detection datasets. Zubiaga et al. (2018) provide a fake news characterization and an overview of detection methods and resolution techniques, focusing on a specific type of disinformation, namely rumor. They also review six datasets for rumor detection. Another survey by Sharma et al. (2019) reviews existing methods and techniques applicable to both detection and mitigation tasks and provides a list of 23 fake news detection datasets. The literature surveyed by Pierri & Ceri (2019) also encompasses fake news diffusion models as well as detection and mitigation techniques, and an overview of 12 fake news detection datasets. Kumar & Shah (2018) characterize fake news according to the intent of detection and the knowledge and surveyed detection methods based on this characterization, without discussing datasets. The surveys authored by Oshikawa, Qian & Wang (2018), Elhadad, Li & Gebali (2019), and Dutta et al. (2019) focused on reviewing fake news detection methods from different perspectives: the first work addresses natural language processing solutions and reviews nine datasets, the second survey focuses on the types of data and the categories of features used in the detection methods and reviews 17 datasets, while the last work focuses on machine learning methods. Bondielli & Marcelloni (2019) focus on detection methods by analyzing both fake news and rumor detection techniques and surveying 11 datasets. Finally, the most recent survey by Zhou & Zafarani (2020) categorizes fake news detection methods according to a fourfold perspective: knowledge, style, propagation, and source of fake information. It also lists eight datasets for automatic fake news detection.

Our survey differs from related surveys in three ways. First, we discuss how fake news detection datasets can be characterized by analyzing the current literature and we define a new characterization specific for FNDD and composed of eleven characteristics extracted from the existing datasets. Second, we conduct a systematic literature search to identify a comprehensive list of fake news detection datasets that we then analyze according to our FNDD characterization. Third, we focus on the requirements for building reliable datasets.

The contributions of the existing surveys have been categorized in Table 1. As can be seen from the table, our survey focuses predominantly on the evaluation phase of fake news detection.

**Table 1:** Comparison among the contributions of our survey and existing surveys on fake news detection.

Contributions references	Fake news characterization	Fake news detection methods	Fake news diffusion models	Fake news mitigation techniques	Evaluation of fake news detection methods			
					Dataset characterization	Datasets	Dataset requirements	Metrics
Shu et al. (2017)	**√**	**√**				**√** **(4 datasets)**		**√**
Zubiaga et al. (2018)	**√**	**√**		**√**		**√** **(6 datasets)**		
Kumar & Shah (2018)	**√**	**√**				**√** **(17 datasets)**		
Oshikawa, Qian & Wang (2018)		**√**				**√** **(9 datasets)**		
Bondielli & Marcelloni (2019)		**√**				**√** **(11 datasets)**		
Dutta et al. (2019)		**√**						
Sharma et al. (2019)	**√**	**√**		**√**		**√** **(23 datasets)**		
Pierri & Ceri (2019)		**√**	**√**	**√**		**√** **(12 datasets)**		
Elhadad, Li & Gebali (2019)		**√**				**√** **(17 datasets)**		
Zhou & Zafarani (2020)	**√**	**√**				**√** **(8 datasets)**		
**Our survey**					**√**	**√** **(27 datasets)**	**√**	

## Survey methodology

**Figure 1:**
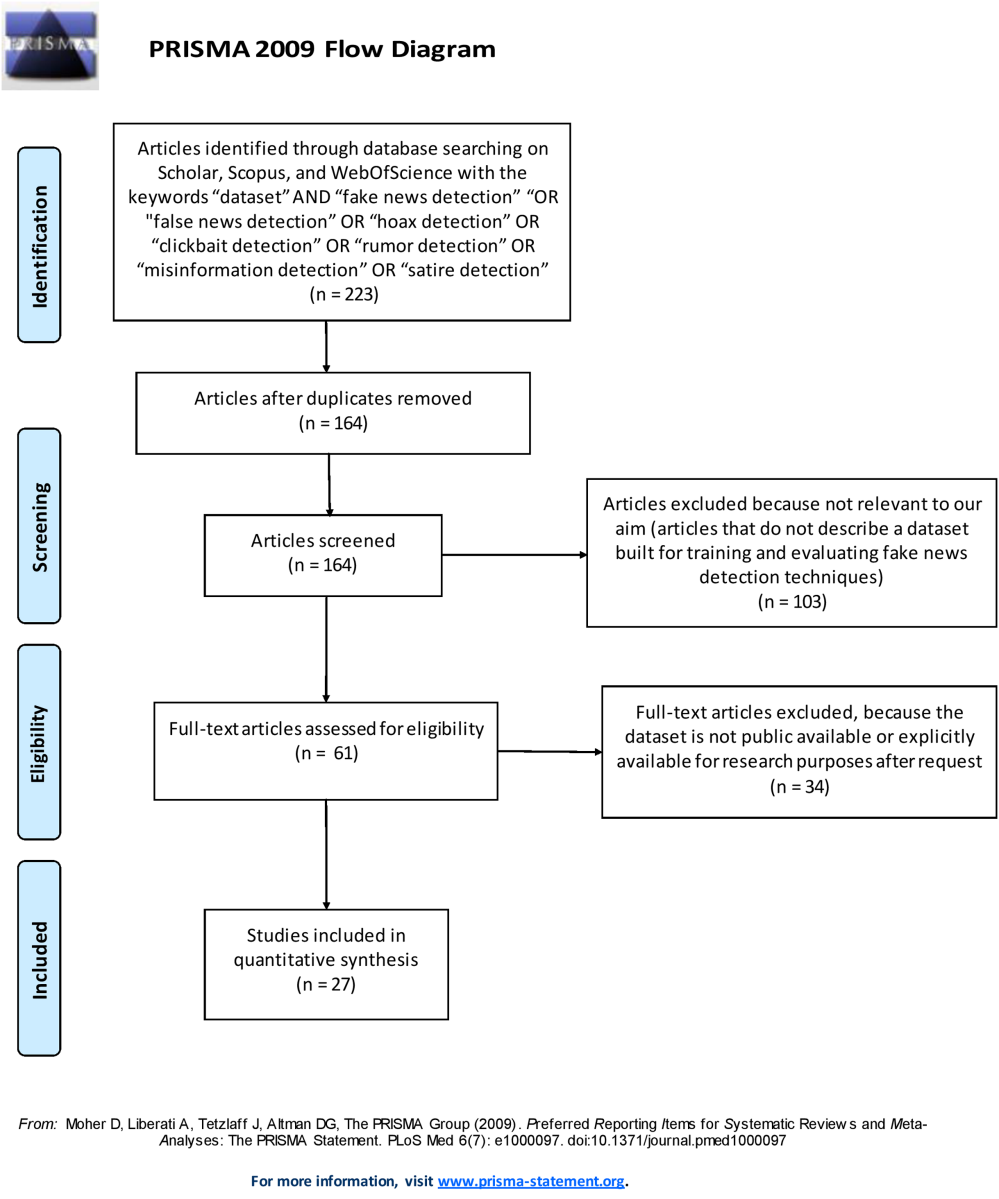
PRISMA 2009 Flow diagram.

This section illustrates the methodology used to select the datasets that have been included in our survey. We conducted a systematic literature search (Brereton et al., 2007; Reyes-Menendez, Saura & Filipe, 2019; De Beer & Matthee, 2021) of the datasets described in scientific papers published from 2000 to 2019 (end of December) that are freely available and identified by searching for scientific articles using three relevant scientific search engines, namely Scholar, Web of Science, and Scopus. Specifically, we followed the systematic literature search process based on the Preferred Reporting Items for Systematic Reviews and Meta-Analyses (PRISMA) recommendations (Moher et al., 2009). The PRISMA recommendations are used as a basis for reporting systematic reviews, as well as for critical appraisal of published systematic reviews. The PRISMA statement includes a four-phase flow diagram (i.e. identification, screening, eligibility, included) that helps improve the reporting of systematic reviews and meta-analyses (see Fig. 1).

The search strings used to select the scientific papers included the following keywords: “dataset” AND “fake news detection” OR “false news detection” OR “hoax detection” OR “clickbait detection” OR “rumor detection” OR “misinformation detection” OR “satire detection”. Only peer-reviewed articles written in English and published in peer-reviewed journals were included in the analysis. A total of 223 articles were returned using these search engines: 155 from Scholar, 43 from Scopus, and 25 from Web of Science, respectively. Excluding any duplicate articles (the same articles retrieved from two different search engines), a total of 164 articles were examined.

To identify the relevant papers from among those retrieved we defined a set of inclusion/exclusion criteria. The first inclusion criterion concerns the relevance to our aim. As we need to include in our survey only studies that describe a dataset built for training and evaluating fake news detection techniques, we read in detail the content of the 164 retrieved papers and extracted 61 datasets discussed in them that fulfill the relevance criterion. The second criterion concerns the public availability of the datasets. This survey extracts and analyzes the characteristics of datasets that are accessible for our investigation. Therefore, only datasets that were either publicly available or explicitly available for research purposes upon request were included in this survey. At the end of this systematic search, 27 datasets were selected and surveyed here. A brief description of each dataset is provided in the Supplemental Article S1.

Figure 1 shows the process followed to identify and select the papers and datasets included in the survey by using the PRISMA 2009 Flow Diagram.

After selecting the datasets included in the survey, a quantitative analysis of the data extracted from them in terms of the frequency distribution of occurrences of their main characteristics (i.e., news domain, application purpose, type of disinformation, language, size, news content, rating scale, spontaneity, media platform, availability and extraction period) is provided (see “Quantitative analysis of surveyed datasets” section).

Finally, a comparative analysis of the datasets is performed using a set of 10 data requirements (availability, verifiability, homogeneity, etc.) that the datasets should satisfy to be fruitfully applied for modeling and testing fake news detection algorithms (see “Comparative analysis of surveyed datasets” section).

### Characteristics of evaluation datasets used in fake news detection

**Figure 2:**
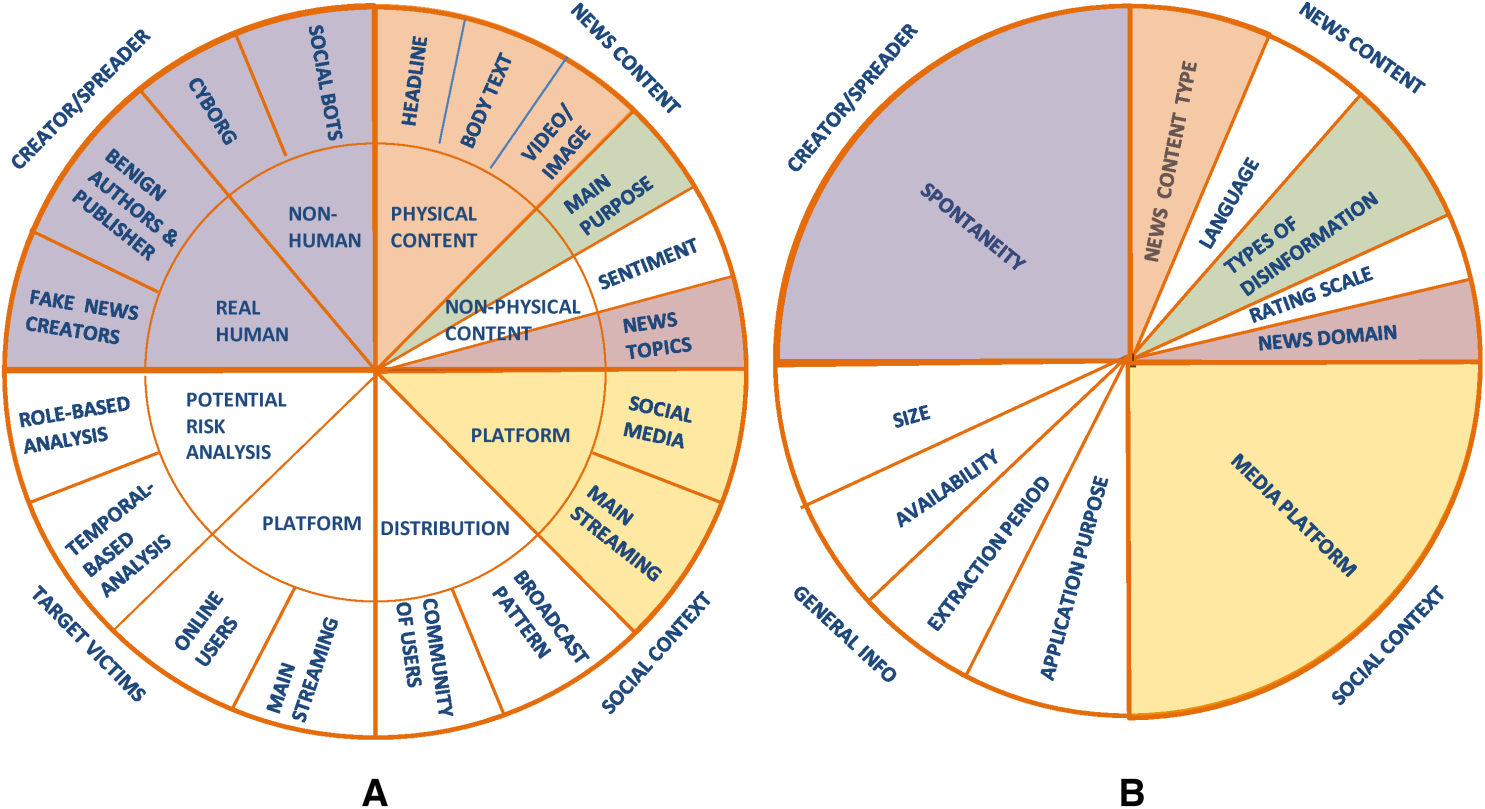
(A) The major components to characterize fake news defined by Zhang & Ghorbani (2019); (B) the major components of the FNDD characterization defined in our survey. The characteristics adapted from Zhang & Ghorbani (2019) are indicated with a colored background, while the newly defined ones are indicated with a white background.

To clearly understand and discriminate among the evaluation datasets for fake news detection, different types of features can be extracted from the descriptions of the 27 surveyed datasets that are described in the Supplemental Article S1. To identify the relevant features that characterize the datasets, we started from the fake news characterization introduced by Zhang & Ghorbani (2019). Their work is of special interest to us since they introduced a clear characterization of online fake news by identifying relevant features related to the users, content, and context that can be adapted to characterize also the datasets used in fake news detection. Specifically, Zhang & Ghorbani (2019) defined four major components to characterize fake news (see Fig. 2A), which are:

(a) the creator/spreader, who can be either a real human (benign authors and publishers who publish fake news unintentionally, and fake news creators) or non-human (cyborgs, social bots);

(b) the target victims, who can be users of online social media or other mainstreaming platforms;

(c) the news content, which can be either physical (e.g., headline, body text, video/image) or non-physical (e.g., main purpose, sentiment, news topics);

(d) the social context, which can be related both to the platforms used to spread the news (social media or mainstreaming platforms) and to the distribution pattern (community of users or broadcast pattern).

Having analyzed the fake news characterization by Zhang & Ghorbani (2019), we selected some of their components and extended this with other ones, to fit the purpose of our work to analyze the features of datasets instead of fake news in general.

Specifically, we checked the descriptions of the datasets provided in the 27 papers, retrieved as illustrated in the “Survey methodology” section, with the aim of searching for whether these descriptions include some of the components defined by Zhang & Ghorbani (2019). This analysis showed that the following three components were explicitly mentioned in the descriptions of the 27 datasets (depicted in Fig. 2A with a colored background): the news content, the social context, and the creator/spreader. On the contrary, the target victims component (Zhang & Ghorbani, 2019) cannot be extracted from the descriptions of the datasets as it is never explicitly mentioned.

Specifically, considering the news content (Zhang & Ghorbani, 2019), the descriptions of the 27 surveyed datasets provide information about both the physical content and the non-physical content (main purpose and news topics), while very few datasets collect information about the sentiment polarity of the news (i.e., positive, negative, neutral). Consequently, our FNDD characterization will include the following three features in the news content component: *News content type*, *type of disinformation*, and *news domain* (depicted in Fig. 2B with a colored background). The *type of disinformation* and the *news domain* correspond, respectively, to the main purpose and news topics in the characterization of Zhang & Ghorbani (2019). We changed their names to better represent what the authors of the 27 surveyed datasets refer to. In addition to these characteristics, we added two more features to this component (i.e., the *language* of the news and the *rating scale* used to assess its truthfulness) since the authors of the 27 datasets describe the datasets also according to these features and we considered it relevant to characterize them.

Turning to the social context (Zhang & Ghorbani, 2019), the aforementioned descriptions provide information about the *media platforms* (e.g., Facebook, Twitter) used to share and spread the news collected in the datasets, while no information about how the news is distributed is given. Therefore, we considered only one feature in this component referring to the media platform.

Finally, referring to the creator/spreader (Zhang & Ghorbani, 2019), the authors of the 27 datasets provide information about the *spontaneity* of the fake news editing (i.e., if fake news items are collected without editing them or have been produced by manipulating real ones). Therefore, we considered only one feature in this component referring to the *spontaneity*.

In addition to that information, the authors of the 27 datasets mentioned four further features of the datasets that are not addressed by Zhang & Ghorbani (2019); these are the *size* of the dataset, the *application purpose* in building the dataset, the online *availability* of the dataset and the *extraction period* when the data have been collected. Consequently, we added a new component to our FNDD characterization, named *general information*, which refers to the datasets' general characteristics, including the *size*, the *application purpose*, the *availability*, and the *extraction period*.

Based on these considerations, we evolve the characteristics proposed by Zhang & Ghorbani (2019) further by proposing the characterization of the datasets of fake and trustful news depicted in Fig. 2B and described below. The re-arranged characteristics are depicted with a colored background, while the newly defined ones are indicated with a white background.

Specifically, our FNDD characterization includes the eleven dataset characteristics described below:

- *News domain*: the dataset can contain fake news items that target certain news domains, such as health, education, tourism, sport, economy, security, science, IT, and political election.

- *Application purpose*: datasets can be built for different aims, such as fake detection, fact-checking, veracity classification, and rumor detection. The first consists of the prediction of the chances of a particular piece of information (news article, reviews, posts, etc.) being intentionally deceptive. Fact-checking refers to the process of vetting and verifying factual statements contained in a piece of information; unlike fake detection, fact-checking works at the level of a particular statement or claim. Veracity classification is very similar to fake detection but attempts to predict the actual truth of a given piece of information. Finally, rumor detection tries to distinguish between verified and unverified information (instead of true or false) and the unverified information may turn out to be true or false, or may remain unresolved.

- *Type of disinformation*: fake news or misleading news may be categorized as fake reviews, fake advertisements, or fake news articles according to the types of false information they contain. Fake news articles can be further classified into (a) hoaxes, considered to be false information deliberately fabricated to masquerade as the truth (Sharma et al., 2019); (b) rumors, which refers to unsubstantiated claims that are disseminated without any evidence to support them (Sharma et al., 2019); and (c) satire, which uses humor, irony, exaggeration, ridicule, and false information to present the news.

- *Language*: this concerns the language of the fake news contained in the dataset, which can be written in different languages according to the sources used to retrieve them. The dataset can be categorized as multi-lingual or monolingual.

- *Size*: the size of the dataset is commonly determined by the number of news items that it contains. It can also be measured in kilobytes/megabytes of the overall archive.

- *News content type:* this concerns linguistic and syntactic features, such as headlines and body text, as well as images and videos of the news. This survey considers the following four types of news content (as they characterize the 27 datasets): headlines, body text, images, and videos.

- *Rating scale*: this concerns the labels that are associated with the news contained in the datasets and used to rate the truthfulness of the news. Different rating scales can be used containing a different number of rating levels. For instance, a five-point rating scale can have the following labels: true, mostly true, mixture of true and false, mostly false, and false. An example of a three-point rating scale is true, false, or unverified.

- *Media platform*: this concerns the digital environment where the news collected in the dataset is shared and spread to the audience. Two main types of media platforms can be used to share and transfer fake news: (i) mainstream media, meaning the traditional media, such as newspapers, TV, and radio, and (ii) online social media, such as Twitter, Facebook, Instagram, and blogs. In this paper, mainstream media include also traditional media (e.g., NBC News, Washington Post, etc.) that have extended the way they spread information from mainstream platforms also to digital platforms.

- *Spontaneity*: fake news in the dataset may be spontaneous if it is automatically extracted from public web sources without editing, or artificial if it is manually generated by asking someone to produce items by manipulating real ones.

- *Availability:* this concerns free online availability of the data contained in the dataset.

-*Extraction period:* this concerns the definition of a specific time frame during which the data have been collected.

A summary of the datasets selected for further analysis in the present review, along with their main characteristics, is shown in Table 2. A brief description of each identified dataset is provided in the Supplemental Article S1.

**Table 2:** Characteristics of the surveyed datasets for fake news detection.

Dataset	News domain	Application purpose	Type of disinformation	Language	Size	News content type	Rating scale	Media platform	Spontaneity	Availability	Extraction time
Yelp dataset (Barbado, Araque & Iglesias, 2019)	Technology	Fake detection	Fake reviews	English	18912 reviews	Text	2 values	Mainstream	Yes	Yes	No
PHEME dataset (Zubiaga et al., 2016)	Society, politics	Rumor detection	Rumors	English and German	330 rumorous conversations and4842 tweets overall	Text	3 values	Social media (Twitter)	Yes	Yes	No
CREDBANK (Mitra & Gilbert, 2015)	Society	Veracity classification	Rumors	English	60 million streaming tweets	Text	5 values	Social media (Twitter)	Yes	Yes	Yes (October 2014 - February 2015)
BuzzFace (Santia & Williams, 2018)	Politics, society	Veracity classification	Fake news articles	English	2263 news	Text	4 values	Social media (Facebook)	Yes	Yes	Yes (September 2016)
FacebookHoax (Tacchini et al., 2017)	Science	Fake detection	Hoaxes	English	15500 posts	Text	2 values	Social media (Facebook)	Yes	Yes	Yes (July 2016 - December 2016)
LIAR (Wang, 2017)	Politics	Fake detection	Fake news articles	English	12836 short statements	Text	6 values	Mainstream + social media (Facebook, Twitter)	Yes	Yes	Yes (2007-2016)
Fact checking dataset (Vlachos & Riedel, 2014)	Politics, society	Fact checking	Fake news articles	English	221 statements	Text	5 values	Mainstream	Yes	Yes	No
FEVER (Thorne et al., 2018)	Society	Fact checking	Fake news articles	English	185,445 claims	Text	3 values	Mainstream	No	Yes	No
EMERGENT (Ferreira & Vlachos, 2016)	Society, technology	Rumor detection	Rumors	English	300 claims, and 2,595 associated article headlines	Text	3 values	Mainstream + social media (Twitter)	Yes	Yes	No
FakeNewsNet (Shu et al., 2018)	Society, politics	Fake detection	Fake news articles	English	422 news	Text, images	2 values	Mainstream + social media (Twitter)	Yes	Yes	No
Benjamin Political News Dataset (Horne & Adali, 2017)	Politics	Fake detection	Fake news articles	English	225 stories	Text	3 values	Mainstream	Yes	Yes	Yes (2014-2015)
Burfoot Satire News Dataset (Burfoot & Baldwin, 2009)	Politics, economy, technology, society	Fake detection	Satire	English	4,233 newssamples	Text	2 values	Mainstream	Yes	Yes	No
BuzzFeed News dataset (Horne & Adali, 2017)	Politics	Fake detection	Fake news articles	English	2,283 news samples	Text	4 values	Social media (Facebook)	Yes	Yes	Yes (2016-2017)
MisInfoText dataset (Torabi & Taboada, 2019)	Society	Fact checking	Fake news articles	English	1,692 news articles	Text	4 values for BuzzFeed and 5 values for Snopes	Mainstream	Yes	Yes	No
Ott et al.’s dataset (Ott et al., 2011)	Tourism	Fake detection	Fake reviews	English	800 reviews	Text	2 values	Social media (TripAdvisor)	No	Yes	No
FNC-1 dataset (Riedel et al., 2017)	Politics, society, technology	Fake detection	Fake news articles	English	49972 articles	Text	4 values	Mainstream	Yes	Yes	No
Spanish fake news corpus (Posadas-Durán et al., 2019)	Science, Sport, Economy, Education, Entertainment, Politics, Health, Security, Society	Fake detection	Fake news articles	Spanish	971 news	Text	2 values	Mainstream	Yes	Yes	Yes (January 2018 - July 2018)
Fake_or_real_news (Dutta et al., 2019)	Politics, society	Fake detection	Fake news articles	English	6,337 articles	Text	2 values	Mainstream	Yes	Yes	No
TSHP-17 (Rashkin et al., 2017)	Politics	Fact checking	Fake news articles	English	33,063 articles	Text	6 values for PolitiFact and 4 values for unreliable sources	Mainstream	Yes	Yes	No
QProp (Barrón-Cedeno et al., 2019)	Politics	Fact checking	Fake news articles	English	51,294 articles	Text	2 values	Mainstream	Yes	Yes	No
NELA-GT-2018 (Nørregaard, Horne & Adalı, 2019)	Politics	Fake detection	Fake news articles	English	713000 articles	Text	2 values	Mainstream	Yes	Yes	Yes(February 2018 - November 2018)
TW_info (Jang, Park & Seo, 2019)	Politics	Fake detection	Fake news articles	English	3472 articles	Text	2 values	social media (Twitter)	Yes	Yes	Yes (January 2015 - April 2019
FCV-2018 (Papadopoulou et al., 2019)	Society	Fake detection	Fake news content	English, Russian, Spanish, Arabic, German, Catalan, Japanese, and Portuguese	380 videos and 77258 tweets	Videos, text	2 values	social media (YouTube, Facebook, Twitter)	Yes	Yes	Yes (April 2017 - July 2017
Verification Corpus (Boididou et al., 2018)	Society	Veracity classification	Hoaxes	English, Spanish, Dutch, French	15629 posts	Text, images, videos	2 values	social media (Twitter)	Yes	Yes	Yes (2012-2015)
CNN / Daily Mail summarization dataset (Jwa et al., 2019)	Politics, society, crime, sport, business, technology, health	Fake detection	Fake news articles	English	287000 articles	Text	4 values	Mainstream	Yes	Yes	Yes (April 2007 - April 2015
Zheng et al.’s dataset (Zheng et al., 2018)	Society	Clickbait detection	Clickbait	Chinese	14922 headlines	Text	2 values	Mainstream + social media (Wechat)	Yes	Yes	No
Tam et al.’s dataset (Tam et al., 2019)	Politics, technology, science, crime, fraud and scam, fauxtography	Rumor detection	Rumors	English	1022 rumors and 4 million tweets	Text	2 values	social media (Twitter)	Yes	Yes	Yes (May 2017 - November 2017)

### Quantitative analysis of surveyed datasets

In this section, a quantitative analysis of the 27 surveyed datasets in terms of the eleven characteristics of our FNDD characterization (i.e., news domain, application purpose, type of disinformation, language, size, news content type, rating scale, media platform, spontaneity, availability, and extraction period), is provided by analyzing the frequency distribution of their occurrences.

**Figure 3:**
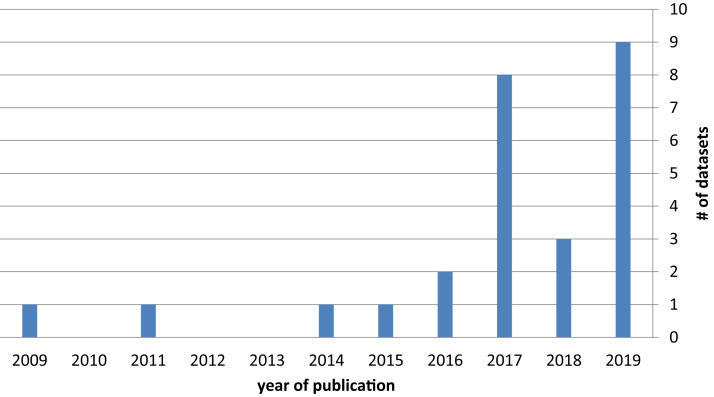
Number of datasets by year of publication.

The temporal distribution of dataset publications, shown in Fig. 3, underscores the increasing interest of the scientific community in the topic of fake news detection datasets, which started growing in 2016 and continues to grow in 2019.

**Figure 4:**
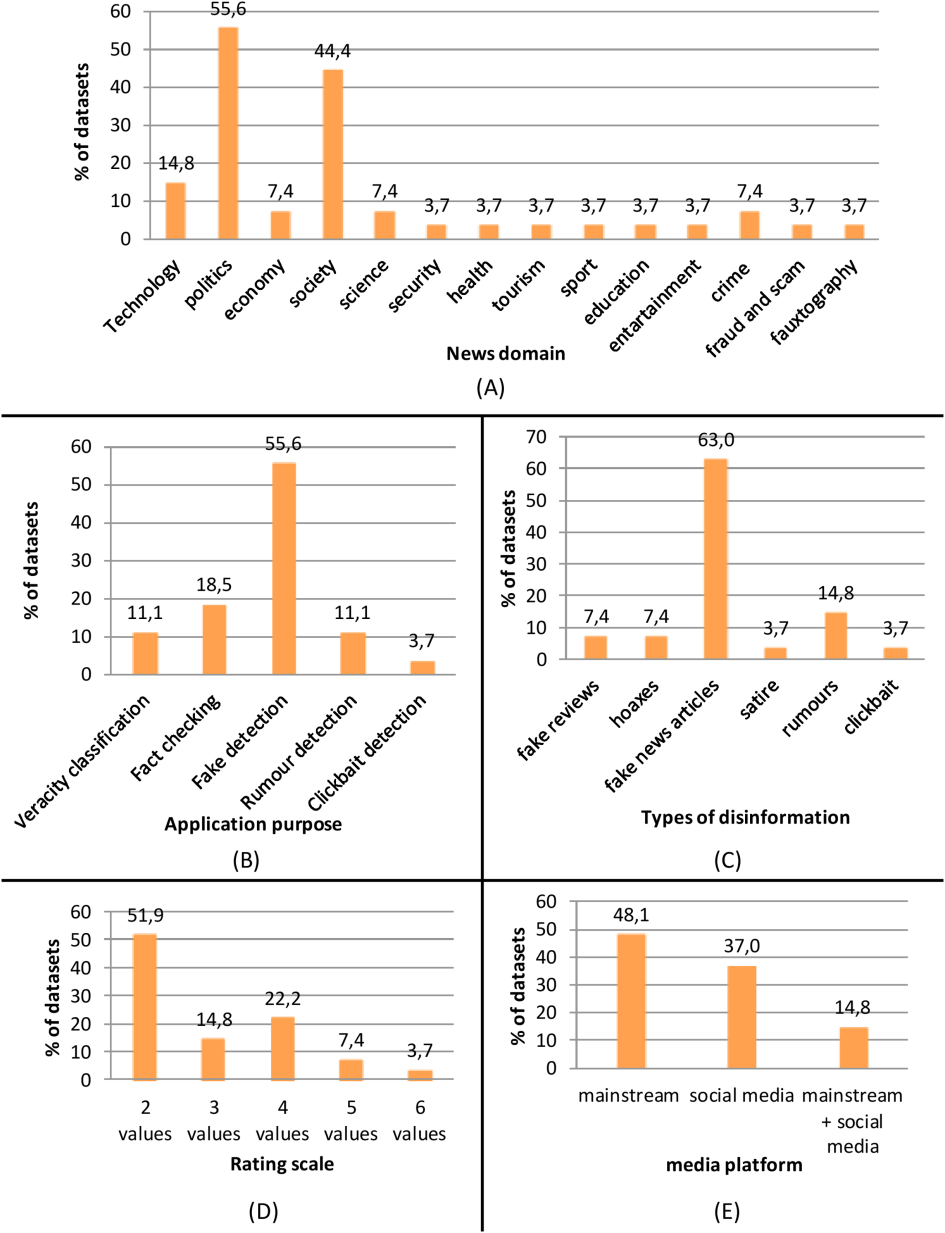
Frequency distribution (percentage) of the following characteristics of the surveyed datasets: (A) news domains; (B) application purpose; (C) types of disinformation; (D) rating scale; (E) media platforms.

Attending to the news domain (see Fig. 4A), the majority of the surveyed datasets collected fake news items concerning politics (55.6%) and society (44.4%), followed by technology (14.8%), economy (7.4%), science (7.4%) and crime (7.4%). Only one dataset collected news on security, health, tourism, sport, education, entertainment, fraud and scam, or fauxtography (3.7%). The high level of attention to the political topic is probably due to the recent studies that demonstrated the significant influence that fake news can exert on political debate (Flynn, Nyhan & Reifler, 2017; Allcott & Gentzkow, 2017), even affecting electoral outcomes during an election cycle. Moreover, a study conducted by Vosoughi, Roy & Aral (2018) founds that political fake news reaches more people and is more viral than false information in any other domain.

The most frequent application purpose of the surveyed datasets is fake detection (55.6%), followed by fact-checking (18.5%), veracity classification (11.1%) and rumor detection (11.1%), and clickbait detection (3.7%) as depicted in Fig. 4B. Fake detection is the most general application purpose and includes the whole process of classifying any types of false information as true or false; this generality makes it the most common area when collecting datasets of fake information. Fact-checking is also quite widely applied, although it is more specific than fake detection as it focuses on the specific step of the fake detection process that consists of collecting the relevant combination of facts related to a claim from different web sources to assign a truth value to the claim. Rumor detection and veracity classification have a more specific and limited task than fake detection, as the former is devoted to determining information that is spreading but yet to be verified (rumor) and the latter aims to determine whether a detected rumor can be classified as true, false, or still unverified. Their specificity and limited scope are the main reasons for which rumor detection and veracity classification are applied less than fake detection. Similarly, clickbait detection has a specific and limited task since it is devoted to determining the use of sensational headlines that attract users to click but are misleading and provocative.

The type of disinformation contained in the datasets (see Fig. 4C) consists mainly of fake news articles (63%), followed by rumors (14.8%), fake reviews and hoaxes (7.4%), satire and clickbait (3.7%). Fake news articles are the most general type of disinformation that is used mainly to spread false information through news outlets for political or financial gain (Zubiaga et al., 2018). This generality makes fake news articles the most common type of disinformation in the surveyed datasets. As suggested by Wardle (2017), the term fake news is being used to refer to different types of disinformation, including also satire, hoaxes, and rumors, which are, by their nature, more specific and, consequently, less frequently addressed by fake news detectors.

Considering the language, 22 of the 27 described datasets are monolingual with news in English (81.5%), while the Spanish fake news corpus is monolingual in Spanish, and Zheng et al. (2018) dataset is monolingual with news in Chinese. Only three datasets (11.1%) are multi-lingual. English, indeed, is the primary language of the Web, and a lot of linguistic tools and fake detection methods have been developed for the English language. Consequently, also the data collected for fake news detection are predominantly written in English.

**Figure 5:**
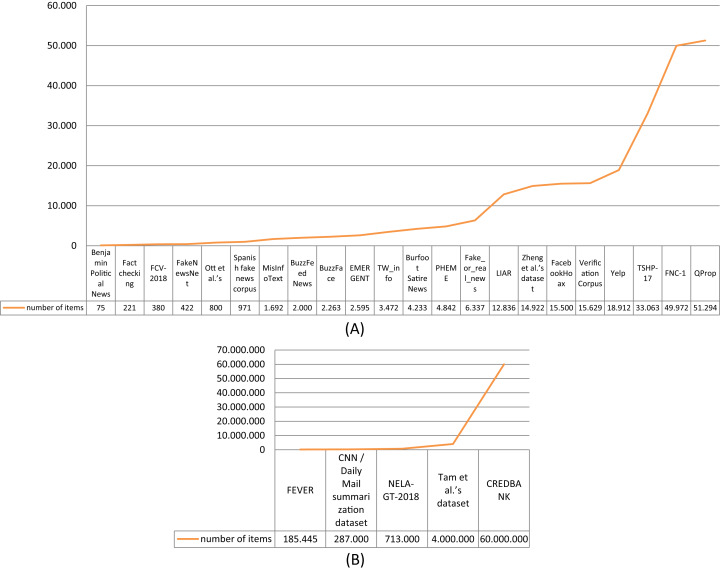
Size (number of items) of the datasets. (A) Size of the surveyed datasets with less than 100,000 collected items; (B) size of the surveyed datasets with more than 100,000 collected items.

Considering the size of the surveyed datasets, the total number of news articles or reviews they contain has been taken into account. The graph depicted in Fig. 5A shows this value in ascending order for 22 of the 27 datasets. Five datasets (CREDBANK, Tam et al. (2019) dataset, NELA-GT-2018, CNN/Daily Mail summarization dataset, and FEVER), which have the highest number of collected articles, are depicted in Fig. 5B since we have used a larger scale for the y axis. The size of the dataset plays an important role in ensuring a high accuracy of the fake detection process. In particular, if the dataset is used to train a fake news detection method that is based on machine learning, it is fundamental to have a large dataset because the performance of this kind of method improves as the training dataset size increases (Torabi & Taboada, 2019). The negative aspect is that very large datasets are less reliable using manual annotation due to time consumption and misclassification (Ghiassi & Lee, 2018). This is why the majority of the surveyed datasets are relatively small, with less than 15,000 news articles.

As concerns the type of news content, 24 of the 27 datasets collected only the text of the news (88.9%), while FakeNewsNet collects also the images included in the news, FVC-2018 collects also the videos, and Verification Corpus collects both images and videos in addition to the text. This fact is probably due to the widespread use of deception detection methods that use natural language processing techniques that depend heavily on text data only (refer to Oshikawa, Qian & Wang (2018) and Su et al. (2020) for a comprehensive survey on these techniques).Only recently have researchers started incorporating images by developing multimodal fake news detection methods, although these still suffer from the scarcity of labeled data due to the labor-intensive process of annotating them.

Considering the rating scale (see Fig. 4D), the majority (51.9%) of the datasets used a two-point rating scale to rate the truthfulness of the fake news items, followed by a four-point rating scale (22.2%), three-point rating scale (14.8%), five-point rating scale (7.4%), and six-point rating scale (3.7%). The predominant use of the binary classification is due to the higher accuracy obtained by two-point prediction compared to six-point classification, which is over 90% for the former and less than 30% for the latter, as found by Oshikawa, Qian & Wang (2018). This is because classifying False news as Mostly False is considered a mistake like classifying True news as False, so a six-point classification method has a higher probability of making mistakes.

The media platforms used to share and transfer fake news items collected in the surveyed datasets are mainly mainstream media (48.1%), followed by online social media (37%), mainly using Facebook or Twitter, and the integration of these two platforms (14.8%), as depicted in Fig. 4E.

Considering the spontaneity, all the described datasets are spontaneous, except FEVER and Ott et al. (2011) dataset, which are annotated by human annotators and the AMT crowdsourcing service, respectively, to manipulate real news items for generating deceptive ones.

As concerns the availability, the survey included only datasets that were either publicly available or explicitly available for research purposes upon request. In the eligibility step of the PRISMA methodology, described in the “Survey methodology” section, 34 publications were excluded because they described datasets that are not publicly available.

Finally, about half of the datasets (48.1%) were extracted within defined extraction period. This allows the extraction of more homogeneous news with less differentiation and more consistent linguistic features, as Kwon, Cha & Jung (2017) found. To summarize, the datasets can be classified according to the characteristics introduced above. Tables 3–12 provide the clusters obtained by applying these criteria and they can be useful when searching for a dataset having specific characteristics.

**Table 3:** Classification of datasets for fake news detection according to the news domain.

		Dataset	# Of datasets
*News domain*	Technology	Yelp, EMERGENT, Burfoot Satire News, FNC-1, CNN/Daily Mail summarization dataset, Tam et al.’s dataset	6
	Politics	PHEME, BuzzFace, LIAR, Fact checking, FakeNewsNet, Benjamin Political News, Burfoot Satire News, BuzzFeed News, FNC-1, Spanish fake news, Fake_or_real_news, TSHP-17, Qprop, NELA-GT-2018, TW_info, CNN/Daily Mail summarization dataset, Tam et al.’s dataset	17
	Economy	Burfoot Satire News, Spanish fake news, CNN/Daily Mail summarization dataset	3
	Society	PHEME, CREDBANK, BuzzFace, Fact checking, FEVER, EMERGENT, FakeNewsNet, Burfoot Satire News, MisInfoText, FNC-1, Spanish fake news, Fake_or_real_news, FCV-2018, Verification Corpus, CNN/Daily Mail summarization dataset, Zheng et al.’s dataset	16
	Science	FacebookHoax, Spanish fake news, Tam et al.’s dataset	3
	Security	Spanish fake news	1
	Health	Spanish fake news, CNN/Daily Mail summarization dataset	2
	Tourism	Ott et al.’s	1
	Sport	Spanish fake news, CNN/Daily Mail summarization dataset	2
	Education	Spanish fake news	1
	Entertainment	Spanish fake news	1
	Crime	CNN/Daily Mail summarization dataset, Tam et al.’s dataset	2
	Fraud and scam	Tam et al.’s dataset	1
	Fauxtography	Tam et al.’s dataset	1

**Note:**

Dataset characteristics and requirements for comparing datasets.

**Table 4:** Classification of datasets for fake news detection according to the application purpose.

		Dataset	# Of datasets
*Application purpose*	Fake detection	Yelp, FacebookHoax, LIAR, FakeNewsNet, Benjamin Political News, Burfoot Satire News, BuzzFeed News, Ott et al.’s, FNC-1, Spanish fake news, Fake_or_real_news, NELA-GT-2018, TW_info, FCV-2018, CNN/Daily Mail summarization dataset	15
	Fact checking	Fact checking, FEVER, MisInfoText, TSHP-17, Qprop	5
	Veracity classification	CREDBANK, BuzzFace, Verification Corpus	3
	Rumour detection	EMERGENT, PHEME, Tam et al.’s dataset	3
	Clickbait detection	Zheng et al.’s dataset	1

**Table 5:** Classification of datasets for fake news detection according to the language.

		Dataset	# Of datasets
*Language*	Monolingual–English	Yelp dataset, CREDBANK, BuzzFace, FacebookHoax, LIAR, Fact checking, FEVER, EMERGENT, FakeNewsNet, Benjamin Political News, Burfoot Satire News, BuzzFeed News, MisInfoText, Ott et al.’s dataset, FNC-1, Fake_or_real_news, TSHP-17, Qprop, NELA-GT-2018, TW_info, CNN/Daily Mail summarization dataset, Tam et al.’s dataset	22
	Monolingual–Spanish	Spanish fake news	1
	Monolingual–Chinese	Zheng et al.’s dataset	1
	Multi-lingual	PHEME, FCV-2018, Verification Corpus	3

**Table 6:** Classification of datasets for fake news detection according to the type of disinformation.

		Dataset	# Of datasets
*Type of disinformation*	Fake news articles	BuzzFace, LIAR, Fact checking, FEVER, FakeNewsNet, Benjamin Political News, BuzzFeed News, MisInfoText, FNC-1, Spanish fake news, Fake_or_real_news, TSHP-17, Qprop, NELA-GT-2018, TW_info, FCV-2018, CNN/Daily Mail summarization dataset	17
	Fake reviews	Yelp dataset, Ott et al.’s dataset	2
	Satire	Burfoot Satire News	1
	Hoaxes	FacebookHoax, Verification Corpus	2
	Rumours	PHEME, CREDBANK, EMERGENT, Tam et al.’s dataset	4
	Clickbait	Zheng et al.’s dataset	1

**Table 7:** Classification of datasets for fake news detection according to the size.

		Dataset	# Of datasets
*Size*	0–1,000	Benjamin Political News, Fact checking, FakeNewsNet, Ott et al.’s dataset, Spanish fake news	5
	1,000–10,000	MisInfoText, BuzzFeed News, BuzzFace, EMERGENT, Burfoot Satire News, PHEME, Fake_or_real_news, TW_info	8
	10,000–100,000	LIAR, FacebookHoax, Yelp dataset, TSHP-17, FNC-1, Qprop, FCV-2018, Verification Corpus, Zheng et al.’s dataset	9
	100,000–100,000,000	FEVER, NELA-GT-2018, CREDBANK, CNN/Daily Mail summarization dataset, Tam et al.’s dataset	5

**Table 8:** Classification of datasets for fake news detection according to the news content type.

		Dataset	# Of datasets
*News content type*	Text	Yelp dataset, PHEME, CREDBANK, BuzzFace, FacebookHoax, LIAR, Fact checking, FEVER, EMERGENT, Benjamin Political News, Burfoot Satire News, BuzzFeed News, MisInfoText, Ott et al.’s dataset, FNC-1, Spanish fake news, Fake_or_real_news, TSHP-17, Qprop, NELA-GT-2018, TW_info, CNN/Daily Mail summarization dataset, Tam et al.’s dataset, Zheng et al.’s dataset	24
	Text, images	FakeNewsNet	1
	Text, videos	FCV-2018	1
	Text, images, videos	Verification Corpus	1

**Table 9:** Classification of datasets for fake news detection according to the rating scale.

		Dataset	# Of datasets
*Rating scale*	2 values	Yelp dataset, FacebookHoax, FakeNewsNet, Burfoot Satire News, Ott et al.’s dataset, Spanish fake news, Fake_or_real_news, Qprop, NELA-GT-2018, TW_info, FCV-2018, Verification Corpus, Zheng et al.’s dataset, Tam et al.’s dataset	14
	3 values	PHEME, FEVER, EMERGENT, Benjamin Political News	4
	4 values	BuzzFace, BuzzFeed News, MisInfoText, FNC-1, TSHP-17, CNN/Daily Mail summarization dataset	6
	5 values	CREDBANK, Fact checking	2
	6 values	LIAR	1

**Table 10:** Classification of datasets for fake news detection according to the media platform.

		Dataset	# Of datasets
*Media platform*	Mainstream media	Yelp dataset, Fact checking, FEVER, Benjamin Political News, Burfoot Satire News, MisInfoText, FNC-1, Spanish fake news, Fake_or_real_news, TSHP-17, Qprop, NELA-GT-2018, CNN/Daily Mail summarization dataset	13
	Online social media	PHEME, CREDBANK, BuzzFace, FacebookHoax, BuzzFeed News, Ott et al.’s dataset, TW_info, FCV-2018, Verification Corpus, Tam et al.’s dataset	10
	Mainstream + Online social media	LIAR, EMERGENT, FakeNewsNet, Zheng et al.’s dataset	4

**Table 11:** Classification of datasets for fake news detection according to the spontaneity.

		Dataset	# Of datasets
*Spontaneity*	Spontaneous	Yelp dataset, PHEME, CREDBANK, BuzzFace, FacebookHoax, LIAR, Fact checking, EMERGENT, FakeNewsNet, Benjamin Political News, Burfoot Satire News, BuzzFeed News, MisInfoText, FNC-1, Spanish fake news, Fake_or_real_news, TSHP-17, Qprop, NELA-GT-2018, TW_info, FCV-2018, Verification Corpus, CNN/Daily Mail summarization dataset, Zheng et al.’s dataset, Tam et al.’s dataset	25
	Artificial	FEVER, Ott et al.’s dataset	2

**Table 12:** Classification of datasets for fake news detection according to the extraction period.

		Dataset	# Of datasets
*Extraction period*	Defined	CREDBANK, BuzzFace, FacebookHoax, LIAR, Benjamin Political News, BuzzFeed News, Spanish fake news, NELA-GT-2018, TW_info, FCV-2018, Verification Corpus, CNN/Daily Mail summarization dataset, Tam et al.’s dataset	13
	Not-defined	Yelp dataset, PHEME, Fact checking, FEVER, EMERGENT, FakeNewsNet, Burfoot Satire News, Ott et al.’s dataset, MisInfoText, FNC-1, Fake_or_real_news, TSHP-17, Qprop, Zheng et al.’s dataset,	14

### Comparative analysis of surveyed datasets

As evidenced in the systematic literature search, many datasets exist and are suitable for evaluating fake news detection methods. It is necessary for researchers to make a comparative analysis of those datasets in order to decide which one to use according to the purpose of their research. To provide this analysis, we need to find parameters on which to compare the characteristics (defined in our FNDD characterization in “Characteristics of evaluation datasets used in fake news detection”) of the datasets described in the Supplemental Article S1.

The main objective of this section is therefore to provide a set of dataset requirements for comparing dataset characteristics and to show how each of the surveyed datasets scores against them. To do this, we need to establish which types of requirements are treated by each dataset.

**Figure 6:**
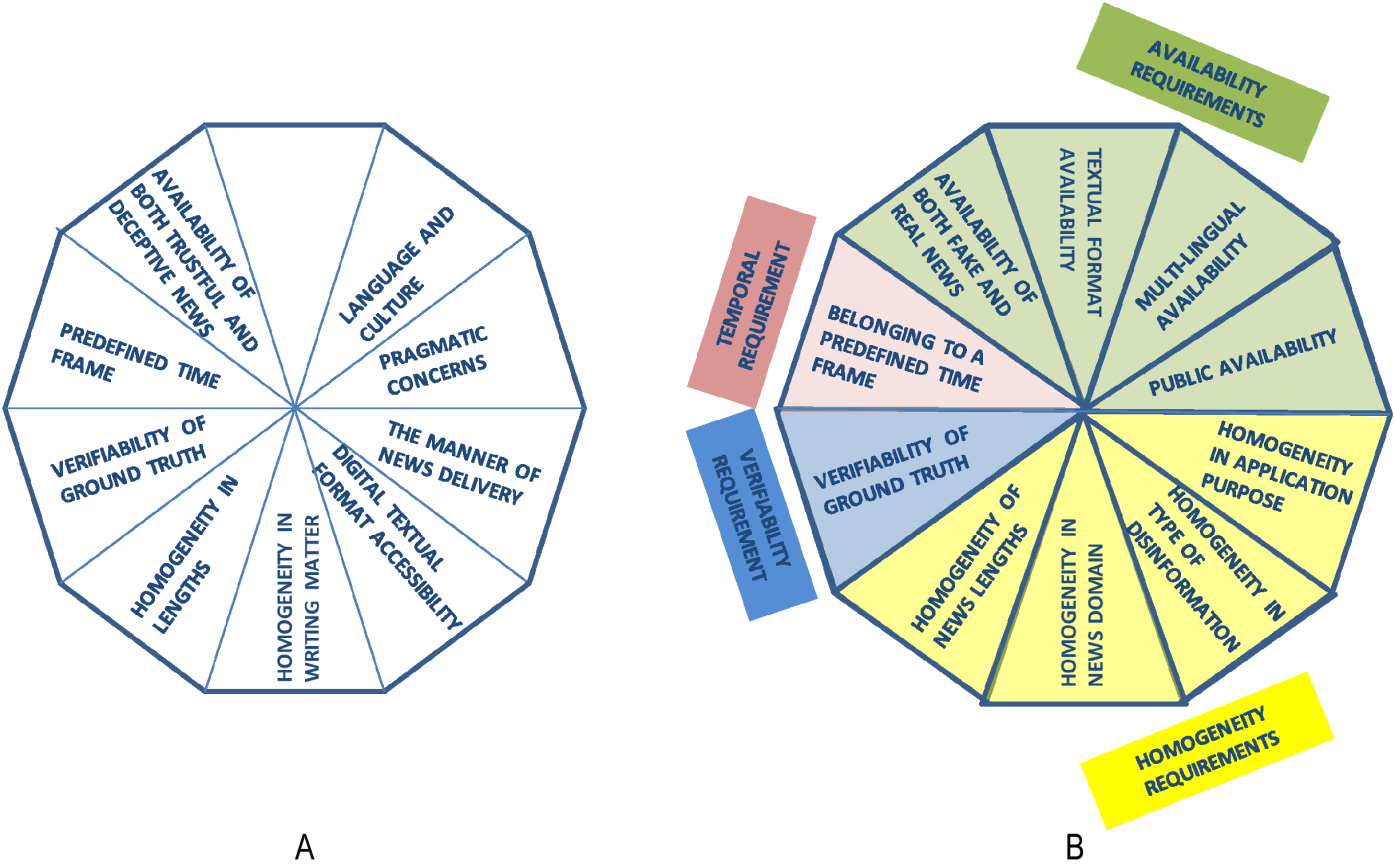
(A) Requirements for fake news detection datasets defined by Rubin, Chen & Conroy (2015): (B) requirements for fake news detection datasets defined in our study.

With this aim, we started from a set of nine requirements defined by Rubin, Chen & Conroy (2015) (see Fig. 6A) that fake news detection datasets should satisfy if they are to be fruitfully applied for modeling and testing fake news detection algorithms. We extended and re-arranged these requirements to fit all the characteristics defined in our FNDD characterization in the “Characteristics of evaluation datasets used in fake news detection” section. As shown in Table 13, indeed, the matching between Rubin et al.’s requirements and our dataset characteristics resulted in several characteristics that are not addressed in the requirements (i.e., type of disinformation, news domain, application purpose). We therefore added new requirements that address these missing features, as shown in Table 14.

**Table 13:** Matching between the Rubin et al.’s requirements and our dataset characteristics.

Rubin et al. requirements	Dataset characteristics according to our FNDD characterisation
Homogeneity in lengths	Media platform
-	Type of disinformation
-	News domain
-	Application purpose
Availability of both trustful and deceptive news	Rating scale
Digital textual format accessibility	News content type
Pragmatic concerns	Availability
Language and culture	Language
Verifiability of ground truth	Spontaneity
Predefined time frame	Extraction period
Homogeneity in writing matter	**-**
The manner of news delivery	**-**

**Table 14:** FNDD characteristics and our requirements for comparing datasets.

Categories of requirements	Our dataset requirements	Dataset characteristics according to our FNDD characterisation
Homogeneity requirements	homogeneity in news lengths	Media platform
	homogeneity in type of disinformation	Type of disinformation
	homogeneity in news domain	News domain
	homogeneity in application purpose	Application purpose
Availability requirements	Fake/real availability	Rating scale
	Textual format availability	News content type
	public availability	Availability
	Multi-lingual availability	Language
Verifiability requirement	verifiability of ground truth	Spontaneity
Temporal requirement	belonging to a predefined time frame	Extraction period

We introduced the following four categories of requirements for classifying the ten requirements considered in our study:

- *Homogeneity requirements*: a dataset is homogeneous if it is made up of news items that are equal or similar to each other according to some characteristics. For example, in the news domain, a dataset is homogeneous if all the news items collected are chosen because they are frogman equal or similar news domain.
- *Availability requirements*: these require that some characteristics associated with the dataset are available. For instance, considering the language, a dataset is multilingually available if the news is collected in more than one language;
- *Verifiability requirements*: these allow the veracity of the news collected in the dataset to be verified;
- *Temporal requirements*: they allow some temporal restrictions over the collected news to be specified.

For each of these categories and each of the characteristics introduced in the “Characteristics of evaluation datasets used in fake news detection” section, we have re-arranged the requirements proposed by Rubin et al. by proposing the set of requirements depicted in Fig. 6B.

A brief description of the ten dataset requirements is provided in the remainder of this section.

1. homogeneity of news length: this implies having datasets with news items that are comparable in length;
2. homogeneity in news domain: this implies having a corpus with texts that are aligned with the topics (e.g., science, government, technology);
3. homogeneity in type of disinformation: this implies having a corpus with texts that are aligned with the type of disinformation (e.g. fake reviews, rumors, fake articles, etc.);
4. homogeneity in application purpose: the aim for which the dataset has been created (e.g., rumor detection, fake deception, fact-checking, etc.) supports interpretation of the situation;
5. availability of both fake and real news: the availability of both fake and real news allows algorithms to find patterns and regularities and, therefore, to improve the identification rate;
6. textual format availability: the availability of texts and textual transcriptions of audio and video is preferred when using algorithms based on natural language processing;
7. public availability: the corpus is publicly available;
8. multi-lingual availability: the availability of news written in multiple languages;
9. verifiability of ground truth: the ability to verify if the news is clearly genuine or fabricated;
10. belonging to a predefined time frame: this implies that the dataset has been collected within a set timeframe.

In Table 15, the public availability requirement is the criterion for selecting the surveyed datasets, while the temporal requirement concerns the period when the data of the datasets are collected; these are relevant in giving more information about the context and the historical period of the considered dataset.

**Table 15:** Analysis of the surveyed datasets for fake news detection according to our requirements.

Dataset	Availability of fake and real news	Textual format availability	Verifiability of ground-truth	Homogeneity of news length	Homogeneity in type of disinformation	Homogeneity in news domain	Belonging to a predefined time frame	Homogeneity in application purpose	Public availability	Multi-lingual availability
Yelp dataset	**√**	**√**	**√**	**√**	**√**	**√**	**-**	**√**	**√**	–
PHEME dataset	**√**	**√**	**√**	**√**	**√**	**-**	**-**	**√**	**√**	**√**
CREDBANK	**√**	**√**	**√**	**√**	**√**	**√**	**√**	**√**	**√**	–
BuzzFace	**√**	**√**	**√**	**√**	**√**	**-**	**√**	**√**	**√**	–
FacebookHoax	**√**	**√**	**√**	**√**	**√**	**√**	**√**	**√**	**√**	–
LIAR	**√**	**√**	**√**	**√**	**√**	**√**	**√**	**√**	**√**	–
Fact checking dataset	**√**	**√**	**√**	**√**	**√**	**-**	**-**	**√**	**√**	–
FEVER	**√**	**√**	**√**	**√**	**√**	**√**	**-**	**√**	**√**	–
EMERGENT	**√**	**√**	**√**	**√**	**√**	**-**	**-**	**√**	**√**	–
FakeNewsNet	**√**	**√**	**√**	**√**	**√**	**-**	**-**	**√**	**√**	–
Benjamin Political News Dataset	**√**	**√**	**√**	**√**	**√**	**√**	**√**	**√**	**√**	–
Burfoot Satire News Dataset	**√**	**√**	**√**	**√**	**√**	**-**	**-**	**√**	**√**	–
BuzzFeed News dataset	**√**	**√**	**√**	**√**	**√**	**√**	**√**	**√**	**√**	–
MisInfoText dataset	**√**	**√**	**√**	**√**	**√**	**√**	**-**	**√**	**√**	–
Ott et al.’s dataset	**√**	**√**	**√**	**√**	**√**	**√**	**-**	**√**	**√**	–
FNC-1 dataset	**√**	**√**	**√**	**√**	**√**	**-**	**-**	**√**	**√**	–
Spanish fake news corpus	**√**	**√**	**√**	**√**	**√**	**-**	**√**	**√**	**√**	**-**
Fake_or_real_news	**√**	**√**	**√**	**√**	**√**	**-**	**-**	**√**	**√**	**-**
TSHP-17	**√**	**√**	**√**	**√**	**√**	**√**	**-**	**√**	**√**	**-**
QProp	**√**	**√**	**√**	**√**	**√**	**√**	**-**	**√**	**√**	**-**
NELA-GT-2018	**√**	**√**	**√**	**√**	**√**	**√**	**√**	**√**	**√**	**-**
TW_info	**√**	**√**	**√**	**√**	**√**	**√**	**√**	**√**	**√**	**-**
FVC-2018	**√**	**√**	**√**	**-**	**√**	**√**	**√**	**√**	**√**	**√**
Verification Corpus	**√**	**√**	**√**	**-**	**√**	**√**	**√**	**√**	**√**	**√**
CNN/Daily Mail summarization dataset	**√**	**√**	**√**	**√**	**√**	*-*	**√**	**√**	**√**	–
Zheng et al.’s dataset	**√**	**√**	**√**	**√**	**√**	**√**	*-*	**√**	**√**	–
Tam et al.’s dataset	**√**	**√**	**√**	**-**	**√**	*-*	**√**	**√**	**√**	–

According to the requirements described above, we have checked the fulfillment of those requirements for each dataset. Table 15 provides the results of our comparison.

As Table 15 underlines, none of the surveyed datasets satisfies all the considered requirements. The BuzzFace, LIAR, Benjamin Political News, BuzzFeed News, NELA-GT-2018 and TW_info datasets satisfy all the cited requirements except for multilinguality. PHEME, FVC-2018, and Verification Corpus are the only three multi-lingual databases since they include: conversational threads in English and German; in English, Russian, Spanish, Arabic, and German; and in English, Spanish, Dutch, French, respectively. PHEME satisfies all the considered requirements except for the timeframe because the corpus has not been collected within a defined timeframe. Meanwhile, FCV-2018 and Verification Corpus satisfy all the considered requirements except for the homogeneity in news length because they contain tweets, images, and videos.

Six of the ten requirements are satisfied by all the analyzed datasets, namely: availability of both fake and real news, textual format availability, verifiability of ground-truth, homogeneity in type of disinformation, homogeneity in application purpose, and public availability.

Table 15 underlines that 13 of the 27 surveyed datasets cover a predefined time frame, while the others have been extracted according to the application purpose, as Table 16 specifies.

**Table 16:** Analysis of the surveyed datasets for fake news detection according to the requirement to belong to a predefined time frame.

		Dataset
*Belonging to a predefined time frame*	*From October 2014 to the end of February 2015*	CREDBANK
	*seven weekdays in September 2016*	BuzzFace
	*2007–2016*	LIAR
	*2014–2015*	Benjamin Political News Dataset
	*from July 2016 to December 2016*	FacebookHoax dataset
	*2016–2017*	BuzzFeed News dataset
	*from January to July of 2018*	Spanish fake news corpus
	*form February 2018 to November 2018*	NELA-GT-2018
	*From January 2015 to April 2019*	TW_info dataset
	*From April 2017 to January 2018*	FCV-2018
	*2012–2015*	Verification Corpus
	*From April 2007 to April 2015*	CNN/Daily Mail summarization dataset
	*From May 2017 to November 2017*	Tam et al.’s dataset

**Figure 7:**
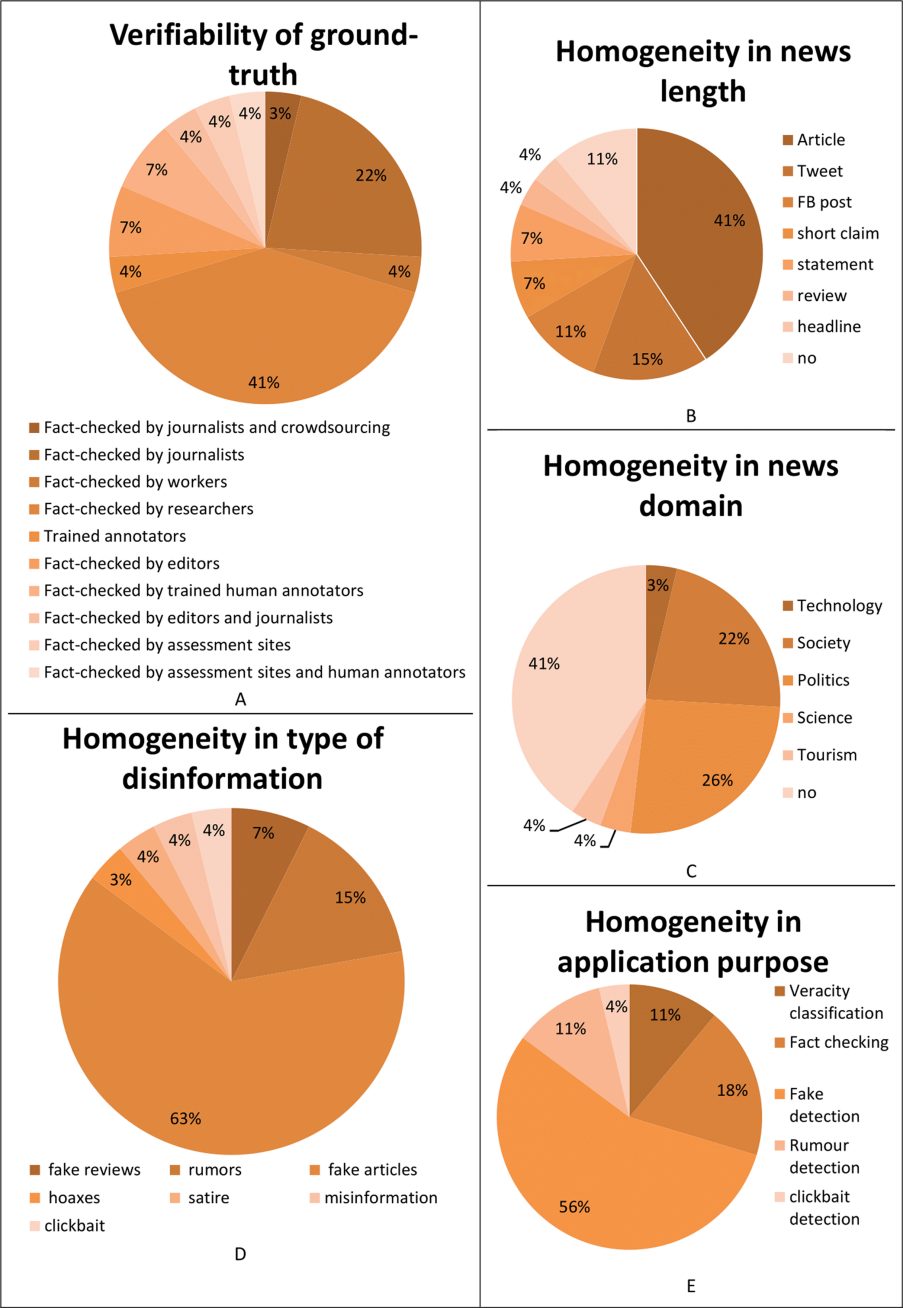
Analysis of the surveyed datasets according to: (A) the verifiability of ground-truth; (B) the homogeneity of the news length; (C) the homogeneity in the news domain; (D) the homogeneity in the type of disinformation; (E) the homogeneity in application purpose.

Analyzing the requirement forth verifiability of ground-truth in Table 17, the majority of the surveyed datasets are fact-checked by researchers (41%), followed by journalists (22%), editors (7%), trained annotators, both human (7%) and artificial (4%), editors and journalists (4%), workers (4%), assessment sites (4%), assessment sites and human annotators (4%), and by journalists and crowdsourcing (3%). This underlines that most of the authors of the surveyed databases directly verified if the news is genuine or fabricated, as Fig. 7A underlines.

**Table 17:** Analysis of the surveyed datasets for fake news detection according to the verifiability of ground-truth requirement.

		Dataset	# Of datasets
Verifiability of ground truth	*Fact-checked by journalists and crowd-sourcing*	Yelp dataset	1
	*Fact-checked by journalists*	PHEME dataset, EMERGENT, Benjamin Political News Dataset, BuzzFeed News dataset, Spanish fake news corpus	5
	*Fact-checked by workers*	CREDBANK, BuzzFace	2
	*Fact-checked by researchers*	FacebookHoax, Fact checking dataset, Burfoot Satire News Dataset, MisInfoText dataset, FNC-1 dataset, Fake_or_real_new, TSHP-17, QProp, TW_info dataset, Zheng et al.’s dataset, Tam et al.’s dataset	11
	*Fact-checked by editors and journalists*	LIAR	1
	*Trained annotators*	FEVER	1
	*Fact-checked by editors*	FakeNewsNet, Verification Corpus	2
	*Fact-checked by trained human annotators*	Ott et al.’s dataset, CNN/Daily Mail summarization dataset	2
	*Fact-checked by assessment sites*	NELA-GT-2018	1
	*Fact-checked by assessment sites and human annotators*	FCV-2018	1

Considering the homogeneity in news length requirement in Table 18, the majority of the surveyed datasets are composed of articles (41%), while 15% of them are composed of Tweets and 11% by Facebook (FB) posts, followed by short claims (7%), statements (7%), reviews (4%), and headlines (4%). 11% of the surveyed datasets do not contain data with homogeneous news length, as Fig. 7B shows.

**Table 18:** Analysis of the surveyed datasets for fake news detection according to the homogeneity in news length requirement.

		Dataset	# Of datasets
News length	*Tweet*	PHEME dataset, CREDBANK, Benjamin Political News Dataset, TW_info dataset	4
	*FB post*	BuzzFace, FacebookHoax, BuzzFeed News dataset	3
	*short claim*	LIAR, FEVER	2
	*statement*	Fact checking dataset, EMERGENT	2
	*article*	FakeNewsNet, Burfoot Satire News Dataset, MisInfoText dataset, FNC-1 dataset, Spanish fake news corpus, Fake_or_real_news, TSHP-17, QProp, NELA-GT-2018, CNN/Daily Mail summarization dataset	10
	*review*	Ott et al.’s dataset	1
	*headline*	Zheng et al.’s dataset	1
	*no*	Yelp dataset, FCV-2018, Verification Corpus, Tam et al.’s dataset	4

Figure 7C makes clear that most of the surveyed datasets do not satisfy the requirements of homogeneity in the news domain (41%), or are collected for a political purpose (26%), followed by societal (22%), tourism (4%), scientific (4%), and technology (3%) purposes.

Considering the homogeneity in the type of disinformation requirement in Table 19, Fig. 7D shows that most of the surveyed datasets have been created with fake articles (63%), followed by rumors (15%), fake reviews (7%), misinformation (4%), satire (4%), clickbait (4%), and hoaxes (3%).

**Table 19:** Analysis of the surveyed datasets for fake news detection according to the homogeneity in type of disinformation requirement.

		Dataset	# Of datasets
Type of disinformation	*fake reviews*	Yelp dataset, Ott et al.’s dataset	2
	*rumors*	PHEME dataset, CREDBANK, EMERGENT, Tam et al.’s dataset	4
	*fake articles*	BuzzFace, LIAR, Fact checking dataset, FEVER, FakeNewsNet, Benjamin Political News Dataset, BuzzFeed News dataset, MisInfoText dataset, Spanish fake news corpus, Fake_or_real_news, TSHP-17, QProp, TW_info dataset, CNN/Daily Mail summarization dataset, FCV-2018, Verification Corpus	15
	*hoaxes*	FacebookHoax, FNC-1 dataset	2
	*satire*	Burfoot Satire News Dataset	1
	*misinformation*	MisInfoText dataset, NELA-GT-2018	2
	*clickbait*	Zheng et al.’s dataset	1

Finally, the datasets are compared considering the homogeneity in the application purpose. Fig. 7E shows that most of the surveyed datasets have been created for fake detection (56%), followed by fact-checking (18%), veracity classification (11%) and rumor detection (11%), and clickbait detection (4%) purposes.

### Open challenges

The quantitative and comparative analyses we have discussed in the previous sections allow us to identify several challenges faced by researchers when defining and developing new datasets for fake news detection.

- *multimedia datasets*: To develop multimedia datasets for fake news detection is one of the major open challenges. Few attempts to incorporate non-textual components of the news, like images and videos, have yet been made. As mentioned before, the only surveyed dataset that collects both images and text is FakeNewsNet. However, it suffers from the scarcity of labeled data (having less than 500 samples) due to the laborious process of annotating them, and this limits its contribution to fake news research. More recently, Nakamura, Levy & Wang (2020) proposed Fakeddit, a large-scale multimodal fake news dataset consisting of over 1 million samples containing text, image, metadata, and comments. Jindal, Vatsa & Singh (2019) also provided a benchmark dataset containing text and images for training and testing models for fake news detection, named NewsBag. These two recent datasets testify to the emerging need to cope with multimedia data, going beyond natural language processing based-fake news detection that depends on text data only. However, further efforts should be made to expand the modality set when building fake news datasets by adding, for example, also audio and video.

- *multi-lingual datasets*: As emerged from the quantitative analysis, most of the developed datasets are only in the English language, limiting the efficacy of fact-checking in different languages. The opportunity to have multi-lingual fake news datasets helps to detect fake news in languages with lacking resources, broadening the datasets’ applicability to detection methods that are not based on specific languages. There is a lack of multi-lingual and cross-domain datasets collected from multiple sources. As we mentioned before, the only surveyed dataset that is multi-lingual (English and German) is PHEME. More recently, Shahi & Nandini (2020) proposed FakeCovid, a multi-lingual dataset containing 5,182 fact-checked news articles on COVID-19 written in 40 different languages. Another multi-lingual dataset has been developed by Abonizio et al. (2020) and contains 9,930 news items written in American English, Brazilian Portuguese, and Spanish. These datasets represent the first attempts to provide a more generic fake news detection approach that is not restricted to any specific language.

- *cross-domain datasets*: The development of cross-domain datasets is another open challenge that emerged from the quantitative analysis discussed in the “Quantitative analysis of surveyed datasets” section. The majority of the surveyed datasets (55%), indeed, collect fake news items concerning only one news domain, which limits the efficacy of news detection methods that suffer from data dependence and are easily affected by noise. Six surveyed datasets (30%) consider only two news domains, while only one dataset (i.e. Spanish fake news corpus) considers nine news domains. More recently, Wang et al. (2020) collected a Weibo dataset containing 7,300 news articles in Chinese across eight domains (health, economic, technology, entertainment, society, military, political, and education). Similarly, Amjad et al. (2020) proposed a benchmark dataset in the Urdu language that contains 900 news articles in 5 different domains (business, health, showbiz, sports, and technology). Therefore, to overcome the limitations on the diversity of fact-checking, future datasets should be collected from a variety of news domains.

- *COVID-19 datasets*: A further consideration regarding the news domain is that after the outbreak of the COVID-19 pandemic there was a proliferation of datasets focused on the health domain. As emerged from the quantitative analysis, indeed, the majority of the surveyed datasets, which were selected before the pandemic, focused mainly on politics and society, following the necessities that emerged during the period in which the news was collected. From March 2020 to date, however, there was a spread of misinformation related to COVID-19 that caused serious social disruption; consequently, several researchers started to collect datasets of fake medical news about the effect of the COVID-19 pandemic. For instance, Shahi & Nandini (2020) proposed FakeCovid, a dataset containing 5,182 fact-checked news articles for COVID-19. Cui & Lee (2020) collected CoAID, a Covid-19 healthcare misinformation dataset including 3,235 fake and true news articles and 851 social platform posts about COVID-19. In addition, Qazi, Imran & Ofli (2020) collected GeoCoV19, a large-scale geolocated Twitter dataset containing more than 524 million multi-lingual tweets posted over 90 days since February 1, 2020. Another Twitter dataset, named ReCOvery and containing a total of 2,029 news articles and 140,820 tweets on coronavirus published from January to May 2020, was collected by Zhou et al. (2020). The repository provides multimodal information on news articles on coronavirus, including textual, visual, temporal, and network information. Besides, COVID-19-FAKES is a publicly available (https://github.com/mohaddad/COVID-FAKES) and automatically annotated bilingual (Arabic/English) COVID-19 Twitter dataset (Elhadad, Li & Gebali, 2021) that was collected from February 04 to March 10, 2020. The dataset has been annotated using the shared information on the official websites and the official Twitter accounts of the WHO, UNICEF, and UN as a source of reliable information, and the collected COVID-19 pre-checked facts from different fact-checking websites to build a ground-truth database. A further example is COV19Tweets Dataset (Lamsal, 2020), a publicly available dataset (https://ieee-dataport.org/open-access/coronavirus-covid-19-tweets-dataset), that contains more than 310 million COVID-19-specific English language tweets and their sentiment scores. Tweets were collected from 20 March to 17 April 2020 and the number of tweets captured in that period per day, on average, was around 893 k. Therefore, in the last months, the need to detect misinformation related to COVID-19 has rapidly risen, making it urgent to collect specific datasets to evaluate the COVID-19 fake news detection methods.

- *Big datasets*: Building a large-scale fake news benchmark dataset is another open challenge that should be pursued to facilitate further research in this area. The quantitative analysis illustrated above showed that only one dataset (the CREDBANK dataset) collected millions of articles, while the majority of the surveyed datasets are relatively small collections with less than 10,000 news articles. A big dataset is fundamental for achieving a highly accurate fake detection process, mainly for fake news detection methods based on deep neural network models, which need a large dataset because their performance improves as the training dataset size increases. Torabi & Taboada (2019) discussed the necessity to use big data for fake news detection and encouraged researchers in this field to share their datasets and to work together towards a standardized large-scale fake news benchmark dataset.

## Conclusion

Due to the need to have data available for training and validating fake news detection algorithms, building high-quality fake news datasets is challenging. Several researchers have addressed this issue by contributing to the effort of automatically detecting fake news and collecting reliable benchmark datasets of fake and trustworthy news.

This study has sought to illustrate how these datasets are structured, providing an insight into the characteristics of each dataset and comparative analysis among them.

The key contributions resulting from the quantitative and comparative analyses performed on the reviewed datasets for fake news detection considering our FNDD characterization can be summarized as follows.

We systematically review 27 popular datasets for fake news detection in terms of their main characteristics (i.e. news domain, application purpose, type of disinformation, language, size, news content, rating scale, media platform, and spontaneity) by providing the quantitative descriptions of their frequencies. This allowed us to show that: (i) the current datasets collect fake news articles mainly written in English and primarily concerning politics and society; (ii) fake detection is the most general application purpose; (iii) most of the datasets are relatively small with less than 10,000 news articles collecting predominantly the text of the news; (iv) the truthfulness of the fake news items is mainly rated using a two-point scale (true or fake); (v) the majority of the datasets collect news items spontaneously published by human creators on mainstream media platforms.

The main characteristics of the 27 popular datasets reviewed have been compared using ten data requirements. We showed that none of the surveyed datasets satisfies all these requirements, and in particular, only seven of them satisfy all the considered requirements except for multilinguality, because only three of the surveyed datasets are multi-lingual but their corpus has not been collected within a timeframe or they are not homogeneous in news length. However, all of the surveyed datasets include fake and real news that is accessible in textual format and publicly available. In addition, the majority of the datasets has been fact-checked by researchers and is composed of news articles. Finally, the majority do not satisfy the requirements of homogeneity in the news domain. This fact could be connected to the need to have information on different topics that can be used to train the fake news detection tools in order not to specialize them on a single topic but to apply them for heterogeneous purposes.

The open challenges in current research that emerged envisage a need to develop benchmark datasets for fake news detection that are multimedia, multi-lingual, cross-domain, and large-scale. Moreover, since the outbreak of the COVID-19 pandemic the need to detect misinformation related to COVID-19 has made it urgent to collect specific COVID-19 fake news datasets.

## Supplemental Information

10.7717/peerj-cs.518/supp-1 Supplemental Information 1 Evaluation datasets used in fake news detection.

10.7717/peerj-cs.518/supp-2 Supplemental Information 2 Fake news data. All identified articles and the elaboration of data.

## Additional Information and Declarations

### Data Availability

The following information was supplied regarding data availability: All the identified articles and the elaboration of the data are available in the Supplemental File. The data are available at: D’Ulizia, Arianna; Caschera, Maria Chiara; ferri, Fernando; Grifoni, Patrizia (2021): Repository of fake news detection datasets. 4TU. ResearchData. Dataset. DOI 10.4121/14151755.v1
