# Supplementary material for: Fake news detection: a survey of evaluation datasets
Source: PeerJ Comput Sci. 2021 Jun 18;7:e518. doi: 10.7717/peerj-cs.518 (PMC8237334; doi:10.7717/peerj-cs.518)
Supplement: Supplemental Information 1 [file peerj-cs-07-518-s001.docx]

# Evaluation datasets used in fake news detection

In the following sub-sections, a brief description of the twenty-eight identified datasets is provided containing information about the nine characteristics introduced in the review.

## Yelp dataset

The Yelp dataset^[[1]](#footnote-2)^ (Barbado et al., 2019) has been recorded to classify fake reviews in the technology domain based on scraping techniques. The dataset is built by web scraping reviews from social Yelp ([www.yelp.com](https://www.yelp.com/)) in four of the most important USA cities (New York, San Francisco, Los Angeles, and Miami). The Yelp dataset is a spontaneous dataset collected for fake detection, and it is fact-checked by journalists and crowd-sourcing. A total of 9456 fake reviews and 9456 trustful reviews in English have been collected and organized according to two types of features: user-centric (personal profile, reviewing activity, trusting information, and social interactions) and review centric (review text).

## PHEME dataset

The PHEME dataset^[[2]](#footnote-3)^ (Zubiaga et al., 2016; Giasemidis et al., 2018; Bondielli and Marcelloni, 2019; Alkhodair et al., 2019) has been created in 2016 by Zubiaga et al. for rumour detection and veracity classification. The dataset is a collection of 330 Twitter rumour threads (4842 tweets) in text form associated with 9 newsworthy events including the Ferguson unrest, the shootingat Charlie Hebdo, and the shooting in Ottawa. The PHEME dataset is multilingual and spontaneous. Each rumours is annotated by professional journalists with its veracity value using the following three labels: true, false, or unverified. Out of the 330 rumour threads, 159 are true, 68 are false and 103 remained unverified.

## CREDBANK

The CREDBANK dataset^[[3]](#footnote-4)^ (Mitra and Gilbert, 2015; Sowmya and Shankar, 2019; Bondielli and Marcelloni, 2019; Zhang and Ghorbani, 2019*)* is collected in 2015 by Mitra and Gilbert for credibility assessment tasks. This corpus is a crowd-sourced collection of streaming tweets in text form that are tracked between October 2014 and February 2015 from Twitter. The dataset comprises more than 60 million tweets in English grouped into 1049 real-world events on society. The tweets are rumours that are built for veracity classification and annotated by 30 human annotators as events or non-events according to their truthfulness. The truthfulness of tweets has been categorized into the following five rating levels: certainly inaccurate, probably inaccurate, uncertain, probably accurate, and certainly accurate.

## BuzzFace

The BuzzFace dataset^[[4]](#footnote-5)^ (Santia and Williams, 2018; Sowmya and Shankar, 2019; Bondielli and Marcelloni, 2019; Reis et al., 2019) has been collected in 2018 by Santia and Williams to build a gold-standard dataset for veracity assessment of news. The dataset was built by starting from the BuzzFeed News dataset [14], described in Section 4.13, and extending the 2263 Facebook news articles with 1.6 million Facebook comments and replies discussing the news content. This dataset contains the texts of Facebook posts on politics and society that are collected in seven weekdays in September 2016 for veracity classification and annotated by journalists. The categorization of the articles, comments, and replies is the same used in the BuzzFace dataset that uses four rating levels: mostly true, not factual content, mixture of true and false, and mostly false.

## FacebookHoax

The FacebookHoax dataset^[[5]](#footnote-6)^ (Tacchini et al., 2017; Sowmya and Shankar, 2019; Bondielli and Marcelloni, 2019) has been collected in 2017 by Tacchini et al. for automatic hoax detection. The dataset contains 15500 public posts in English from 32 Facebook pages (14 conspiracy and 18 scientific) collected from July 2016 to December 2016 and annotated by researchers. The dataset organizes the texts of the posts into the following two categories: hoaxes and non-hoaxes. A total of 8923 (57.6%) hoax posts and 6577 (42.4%) non-hoax posts have been collected.

## LIAR

The LIAR dataset^[[6]](#footnote-7)^ (Wang, 2018; Bondielli and Marcelloni, 2019; Zhang and Ghorbani, 2019; Aldwairi and Alwahedi, 2018; Torabi and Taboada, 2019) has been collected and published by Wang for online fake news detection in 2017. The dataset comprises 12836 manually labelled short statements in English on politics retrieved from the fact-checking website PolitiFact.com from 2007 to 2016 and fact-checked by editors and journalists. The truthfulness of the statements has been rated according to the following six rating levels: true, mostly true, half true, barely true, false, and pants-fire. In addition to the texts of the statements, the dataset contains further information related to the context/venue and the speaker’s profile metadata (affiliation, state, job, and prior history).

## Fact checking dataset

The fact checking dataset^[[7]](#footnote-8)^ (Vlachos and Riedel, 2014; Bondielli and Marcelloni, 2019) is provided in 2014 by Vlachos and Riedel for assessing the truthfulness of a claim. The dataset contains 221 English statements on politics and society, that are fact-checked by journalists on two the fact-checking websites, i.e PolitiFact.com and Channel4.com. This dataset has been collected for online fake news detection. The truthfulness of statements is rated using a five-point scale, that is: true, mostly true, half true, mostly false, and false. For each statement, the dataset contains the text, the speaker, the time it was made, the label assigned by the journalists, the link to the webpage.

## FEVER

The FEVER dataset^[[8]](#footnote-9)^ (Thorne et al., 2018; Bondielli and Marcelloni, 2019; Torabi and Taboada, 2019) has been generated in 2018 by Thorne et al. to verify textual sources for fact-checking. It consists of 185445 text claims of fake articles on the society domain, which were generated by human annotators that extracted them from Wikipedia and mutated the meaning in a variety of ways, and, finally, classified them as supported, refuted or not_enough_info.

## EMERGENT

The EMERGENT dataset^[[9]](#footnote-10)^ (Ferreira and Vlachos, 2016; Bondielli and Marcelloni, 2019; Torabi and Taboada, 2019) has been collected in 2016 by Ferreira and Vlachos for rumour detection. It contains 300 rumoured text statements on society and technology associated with 2595 articles retrieved from rumour websites, such as snopes.com, and Twitter accounts, such as @Hoaxalizer. The spontaneous articles are annotated by a group of journalists to indicate whether their stance are for, against, or observing the claim. The veracity of claims is judged consequently as true, false, or unverified.

## FakeNewsNet

The FakeNewsNet dataset^[[10]](#footnote-11)^( Shu et al., 2018; Bondielli and Marcelloni, 2019; Zhang and Ghorbani, 2019) is provided by Shu et al. in 2018 with the aim to boost the open research studies related to fake news detection. The dataset is composed of 211 fake news and 211 real news that are collected from two fact-checking websites that are GossipCop.com and PolitiFact.com. These fake news articles are spontaneous texts and images on society and politics domains and they are fact-checked by editors. For each news article, the dataset also contains multi-dimensional information related to news content, social context, and spatiotemporal information.

## Benjamin Political News Dataset

The Benjamin Political News Dataset^[[11]](#footnote-12)^ (Horne and Adali, 2017; Zhang and Ghorbani, 2019; Ahmed et al., 2018) has been created in 2017 by Horne and Adali with the aim of detecting fake online political and satire stories. The dataset contains 225 stories written in English and collected from two lists of mainstream media: the Business Insider’s list of real sources^[[12]](#footnote-13)^ and the Zimdar’s list of fake and satirical news websites^[[13]](#footnote-14)^. The dataset organizes the texts of the news, spontaneously published by human creators, into the following three categories: real, fake and satire (75 stories for each category).

## Burfoot Satire News Dataset

The Burfoot Satire NewsDataset^[[14]](#footnote-15)^ (Burfoot and Baldwin, 2009; Horne and Adali, 2017; Zhang and Ghorbani, 2019) is one of the first created datasets collected by Burfoot and Baldwin in 2009 with the aim of automatically detecting satirical articles dealing with political, economic, technological, and societal news. The dataset contains 4000 real news articles and 233 satire news articles written in English. The real news are collected by randomly sampling them from a mainstream media platform, called English Gigaword Corpus, while the satire news are selected to relate closely to at least one of the real news. The dataset organizes the text of the news, spontaneously published by human creators, into two categories: newswire and satire.

## BuzzFeed News dataset

The BuzzFeed News dataset^[[15]](#footnote-16)^ (Horne and Adali, 2017; Zhang and Ghorbani, 2019) has been recorded in 2017 by Horne and Adali with the aim of detecting fake and satirical political news. The dataset is composed of 2283 news posted spontaneously on the social medium Facebook in September 2016 and found in a Buzzfeed’s article on fake US Presidential election news on Facebook^[[16]](#footnote-17)^. The news, written in English, have been categorized into the following four rating levels: mostly true, not factual content, mixture of true and false, and mostly false. In addition to the text of the news, further information of each news has been recorded in the dataset, such as the post type, the number of shares, the number of reactions, and the number of comments.

## MisInfoText dataset

The MisInfoText dataset^[[17]](#footnote-18)^ (Torabi and Taboada, 2019) has been collected in 2019 by Torabi and Taboada to automatically detect misinformation in news. The dataset is composed of 1692 news articles on societal topics spontaneously published by human creators. The dataset organizes the text of the news, written in English and retrieved from two fact-checking websites that are Buzzfeed.com (1380 news articles) and Snopes.com (312 news articles). The articles retrieved from Buzzfeed.com have been categorized as described in Section 4.13, while three annotators assessed manually the veracity of articles retrieved from Snopes.com and classified them using a five-point scale, that is: [fully] true, mostly true, mixture of true and false, mostly false, and [fully] false.

## Ott et al.’s dataset

The Ott et al.’s dataset^[[18]](#footnote-19)^ (Ott et al., 2011; Viviani and Pasi, 2017; Ahmed et al., 2018) is one of the first publicly available opinion spam datasets created by Ott et al. in 2011 with the aim of detecting deceptive reviews on tourism. The dataset contains the text of 800 positive reviews written in English and organized into the following two categories: deceptive (400 reviews) and truthful (400 reviews). The truthful reviews are retrieved from the social media TripAdvisor.com, while the deceptive reviews are manually generated by using the Amazon Mechanical Turk (AMT) crowdsourcing service.

## FNC-1 dataset

The Fake News Challenge (FNC-1) dataset^[[19]](#footnote-20)^ (Riedel et al., 2017; Aldwairi and Alwahedi, 2018; Thota et al., 2018) has been recorded by Riedel et al. in 2017 for fake detection by assisting humans to detect inaccurate claims. This dataset provides 49972 news articles derived from the Emergent project^^[[20]](#footnote-21)^^ dealing with political, societal, and technological topics. The news, written in English and spontaneously published by human creators, have been categorized into the following four rating levels: agree, disagree, discuss, or unrelated. Out of the about 50000 stances, 3678 agree, 840 disagree, 8909 discuss, and 3654 are unrelated. Each stance is composed of the headline of the news, the body of the news, and the label that identifies if the news headline agrees, disagrees, discusses, or is unrelated to the body of the news.

## Spanish fake news corpus

The Spanish fake news corpus^[[21]](#footnote-22)^ (Posadas-Durán et al., 2019) has been collected for automatic fake news detection in the Spanish language. A total of 971 news (491 true and 480 fake) across nine domains (science, sport, economy, education, entertainment, politics, health, security, and society) were retrieved from January to July of 2018 from several resources on mainstream media platforms. The dataset organizes the text of the news spontaneously published by human creators and manually tagged into two categories (true or fake).

## Fake_or_real_news

The Fake_or_real_news dataset^[[22]](#footnote-23)^ (Dutta et al., 2019) is collected for fake news detection. It consists of a total of 6337 articles written in English and dealing with political and societal topics. The text of the news, spontaneously published by human creators, is retrieved from mainstream media platforms not specified by the authors and is organized in the following two categories: true (50%) and false (50%). For each article, the headline, the body, and the label that identifies if it is true or false are recorded.

## TSHP-17

The TSHP-17 dataset^[[23]](#footnote-24)^ (Rashkin et al., 2017; Barrón-Cedeno, 2019) is provided in 2017 by Rashkin et al. for political fact-checking. The dataset contains 33063 news articles retrieved from two mainstream media sources: 10483 statements are retrieved from the fact-checking website PolitiFact.com and rated using a six-point scale (True, MostlyTrue, Half-true, Mostly False, False, and Pants-on-fire False), while 22580 articles are retrieved from different types of unreliable sources and classified as satire (5750 articles), propaganda (5330 articles), hoaxes (5750 articles) and trusted (5750 articles). The text of the news, spontaneously published by human creators, are collected in the dataset.

## QProp

The QProp dataset^[[24]](#footnote-25)^ (Barrón-Cedeno, 2019) has been recorded with the aim of fact-checking and automatically assessing the level of propagandistic content in articles with a political topic. The dataset is composed of 51294 articles that are retrieved from 104 mainstream media web sources and classified into two categories: propagandistic (5737 articles) and trustworthy (45557 articles). The propaganda and the trustworthiness of all the articles are judged using the Media Bias/Fact Check website. In addition to the text of the news, further information recorded in the dataset concerns geographical information, average sentiment, publication date, author, and official source name.

## NELA-GT-2018

NELA-GT-2018 dataset^[[25]](#footnote-26)^ (Nørregaard et al. 2019) is a political news article data covering 713000 articles in English collected between 02/2018-11/2018 for studying misinformation in news articles. The NELA-GT-2018 articles are collected directly from 194 news and media outlets including mainstream, hyper-partisan, and conspiracy sources. The dataset contains true and false texts and it incorporates ground truth ratings of the sources from 8 different assessment sites (i.e., NewsGuard, Pew Research Center, Wikipedia, OpenSources, Media Bias/Fact Check, AllSides, BuzzFeed News, Politifact) which all attempt to assess the reliability and/or the bias of news and covering multiple dimensions of veracity, including reliability, bias, transparency, adherence to journalistic standards, and consumer trust.

## TW_info

The TW_info dataset^[[26]](#footnote-27)^ (Jang et al., 2019) has been recorded with the aim of identifying the best features of fake news for fake news classification. The dataset contains 3472 news articles with a political topic retrieved by the social media platform Twitter and classified as fake news and real news. In addition to the text of the tweets, further information recorded in the dataset concerns URL information, special characters, emphasised words, and emotion.

## FCV-2018

The Fake Video Corpus 2018^[[27]](#footnote-28)^ (FVC-2018) (Papadopoulou et al., 2019) has been recorded to detect misleading news content. The dataset contains 380 videos with societal topic and their 5195 near-duplicates collected from three social media platforms: YouTube, Facebook, and Twitter. The dataset also includes 77258 tweets with links to the dataset’s videos. The dataset was created incrementally, by starting from 104 videos collected in 2016, and extended to 227 videos collected between April 2017 and July 2017, and finally to 380 videos collected between November 2017 and January 2018. The videos, spontaneously published by human creators, were annotated as fake (200 videos) and real (180 videos) using both debunking sites, such as snopes.com, and human annotators. The most frequent language used in the collected videos is English, followed by Russian, Spanish, Arabic, German, Catalan, Japanese, and Portuguese.

## Verification Corpus

The Verification Corpus^[[28]](#footnote-29)^ (Boididou et al., 2018) has been recorded with the aim of classifying the veracity of multimedia Twitter posts into credible or misleading. The dataset consists of 6225 real and 9404 fake tweets related to 17 events (or hoaxes) with societal topic and comprises 193 real images, 218 fake images, and two fake videos collected from the social media platform Twitter in the period 2012-2015. The tweets are written in four different languages: English, Spanish, Dutch, and French.

## CNN / Daily Mail summarization dataset

## The CNN / Daily Mail summarization dataset^^[[29]](#footnote-30)^^ (Jwa et al., 2019) is collected for fake news detection. It consists of a total of approximately 287000 news articles written in English from two mainstream media sources: CNN and Daily Mail websites. The CNN articles were 90000 (plus 380,000 questions) collected from the period between April 2007 and April 2015, while the Daily Mail articles were 197000 (plus 879000 questions) collected in the period between June 2010 and April 2015. The articles are classified into four categories according to the relation that the body text may have with the headline: Agrees, Disagrees, Discusses, and Unrelated.

## Zheng at al.’s dataset

Zheng at al.’s dataset^[[30]](#footnote-31)^ (Zheng at al., 2017) has been recorded with the aim of clickbait detection. The dataset contains 14922 headlines, that are classified in clickbait or not clickbait. The headlines were entracted from four famous Chinese news websites (Tencent, 163, Sohu, Sina), well-known blogs, popular BBSs and Wechat official accounts. Therefore, the dataset contains four article types: news, blogs, BBSs and Wechats.

## Tam at al.’s dataset

Tam et al.’s dataset^[[31]](#footnote-32)^ (Tam et al., 2019) has been collected for rumour detection. The dataset contains 4 million tweets, 3 million users, 28893 hash tags, and 305115 linked articles, revolving around 1022 rumours from 01/05/2017 to 01/11/2017. The collected data are classified in rumours and not rumours and they are related to different domains: politics rumours that are related to all political issues; fraud and scam rumours that are related to online hoax/scam entreating users to share posts and photographs under the false premise of a greater good; fauxtography rumours that are related to images or videos circulating on the Web; crime rumours that are related to criminology and incidents, such as the Las Vegas shooting; and science and technology rumours that are related to scientific myths and exaggerated technological inventions.

1. The dataset is available on request by email to o.araque@upm.es [↑](#footnote-ref-2)
2. https://figshare.com/articles/PHEME_rumour_scheme_dataset_journalism_use_case/2068650/2 [↑](#footnote-ref-3)
3. https://github.com/compsocial/CREDBANK-data [↑](#footnote-ref-4)
4. https://github.com/gsantia/BuzzFace [↑](#footnote-ref-5)
5. https://github.com/gabll/some-like-it-hoax [↑](#footnote-ref-6)
6. https://www.cs.ucsb.edu/ ̃william/data/liar_dataset.zip [↑](#footnote-ref-7)
7. https://sites.google.com/site/andreasvlachos/resources [↑](#footnote-ref-8)
8. http://fever.ai/resources.html [↑](#footnote-ref-9)
9. https://github.com/willferreira/mscproject [↑](#footnote-ref-10)
10. https://github.com/KaiDMML/FakeNewsNet [↑](#footnote-ref-11)
11. https://github.com/rpitrust/fakenewsdata1 [↑](#footnote-ref-12)
12. <https://www.businessinsider.com/here-are-the-most-and-least-trusted-news-outlets-in-america-2014-10?IR=T> [↑](#footnote-ref-13)
13. <https://docs.google.com/document/d/10eA5-mCZLSS4MQY5QGb5ewC3VAL6pLkT53V_81ZyitM/preview> [↑](#footnote-ref-14)
14. https://github.com/rfong/satire/tree/master/corpus [↑](#footnote-ref-15)
15. https://github.com/BuzzFeedNews/2016-10-facebook-fact-check/tree/master/data [↑](#footnote-ref-16)
16. <https://www.google.com/url?sa=t&rct=j&q=&esrc=s&source=web&cd=1&cad=rja&uact=8&ved=2ahUKEwjlwqP1t5TlAhXSfFAKHT84BdAQFjAAegQIARAB&url=https%3A%2F%2Fwww.buzzfeednews.com%2Farticle%2Fcraigsilverman%2Fviral-fake-election-news-outperformed-real-news-on-facebook&usg=AOvVaw0gSo5Ss3fMr769rr-XDG5h> [↑](#footnote-ref-17)
17. https://github.com/sfu-discourse-lab/MisInfoText [↑](#footnote-ref-18)
18. http://www.cs.cornell.edu/ ̃myleott/op_spam [↑](#footnote-ref-19)
19. https://github.com/uclmr/fakenewschallenge [↑](#footnote-ref-20)
20. http://www.emergent.info/ [↑](#footnote-ref-21)
21. https://github.com/jpposadas/FakeNewsCorpusSpanish [↑](#footnote-ref-22)
22. https://www.kaggle.com/rchitic17/real-or-fake [↑](#footnote-ref-23)
23. https://homes.cs.washington.edu/~hrashkin/factcheck.html [↑](#footnote-ref-24)
24. <http://proppy.qcri.org/about.html> [↑](#footnote-ref-25)
25. <https://dataverse.harvard.edu/dataverse/nela> [↑](#footnote-ref-26)
26. http://www.mdpi.com/2079-9292/8/12/1377/s1 [↑](#footnote-ref-27)
27. https://mklab.iti.gr/results/fake-video-corpus/ [↑](#footnote-ref-28)
28. github.com/MKLab-ITI/image-verification-corpus [↑](#footnote-ref-29)
29. https://github.com/abisee/cnn-dailymail [↑](#footnote-ref-30)
30. https://github.com/chenjinyuan87/cbcnn [↑](#footnote-ref-31)
31. http://tiny.cc/p1s2qy [↑](#footnote-ref-32)
